# Differential Competitive Growth of Transgenic Subclones of Neuroblastoma Cells Expressing Different Levels of Cathepsin D Co-Cultured in 2D and 3D in Response to EGF: Implications in Tumor Heterogeneity and Metastasis

**DOI:** 10.3390/cancers16071343

**Published:** 2024-03-29

**Authors:** Eleonora Secomandi, Andrea Esposito, Giulia Camurani, Chiara Vidoni, Amreen Salwa, Chiara Lualdi, Letizia Vallino, Alessandra Ferraresi, Ciro Isidoro

**Affiliations:** Laboratory of Molecular Pathology, Department of Health Sciences, Università del Piemonte Orientale “A. Avogadro”, Via Solaroli 17, 28100 Novara, Italy; eleonora.secomandi@uniupo.it (E.S.); andrea.esposito@uniupo.it (A.E.); giuliacamurani@gmail.com (G.C.); chiara.vidoni@med.uniupo.it (C.V.); salwa.amreen@uniupo.it (A.S.); chiara.lualdi@uniupo.it (C.L.); letizia.vallino@uniupo.it (L.V.)

**Keywords:** cancer, 3D neurospheres, lysosome, cell adhesion, clonal evolution, metastasis, tumor heterogeneity

## Abstract

**Simple Summary:**

Neuroblastoma is a tumor arising from the sympathetic nervous system, and epidermal growth factor (EGF) influences its growth and metastatic behavior. We demonstrated that cathepsin D (CD) counteracts EGF-induced neuroblastoma cell growth in 2D by downregulating EGFR/MAPK signaling. Aggressive NB is highly metastatic, and whether CD is involved in the survival of metastatic NB clones is not known. Here, we addressed how CD differentially affects cell growth in suspension versus the adherent condition. To reproduce tumor heterogeneity, we co-cultured transgenic clones silenced for or overexpressing CD. We found that the Over CD clone had an advantage for growth in suspension, while the CD knocked-down clone was favored for the adherent growth in 2D. This dual role of CD suggests that clonal evolution may produce subclones with different CD levels, conferring survival and growth advantages depending on the metastatic step. We propose that epigenetic regulation of CD expression could be an additional strategy to prevent NB metastases.

**Abstract:**

Neuroblastoma (NB) is an embryonal tumor arising from the sympathetic central nervous system. The epidermal growth factor (EGF) plays a role in NB growth and metastatic behavior. Recently, we have demonstrated that cathepsin D (CD) contrasts EGF-induced NB cell growth in 2D by downregulating EGFR/MAPK signaling. Aggressive NB is highly metastatic to the bone and the brain. In the metastatic process, adherent cells detach to form clusters of suspended cells that adhere once they reach the metastatic site and form secondary colonies. Whether CD is involved in the survival of metastatic NB clones is not known. Therefore, in this study, we addressed how CD differentially affects cell growth in suspension versus the adherent condition. To mimic tumor heterogeneity, we co-cultured transgenic clones silenced for or overexpressing CD. We compared the growth kinetics of such mixed clones in 2D and 3D models in response to EGF, and we found that the Over CD clone had an advantage for growth in suspension, while the CD knocked-down clone was favored for the adherent growth in 2D. Interestingly, on switching from 3D to 2D culture conditions, the expression of E-cadherin and of N-cadherin increased in the KD-CD and Over CD clones, respectively. The fact that CD plays a dual role in cancer cell growth in 2D and 3D conditions indicates that during clonal evolution, subclones expressing different level of CD may arise, which confers survival and growth advantages depending on the metastatic step. By searching the TCGA database, we found up to 38 miRNAs capable of downregulating CD. Interestingly, these miRNAs are associated with biological processes controlling cell adhesion and cell migration. The present findings support the view that during NB growth on a substrate or when spreading as floating neurospheres, CD expression is epigenetically modulated to confer survival advantage. Thus, epigenetic targeting of CD could represent an additional strategy to prevent NB metastases.

## 1. Introduction

Neuroblastoma (NB) is one of the most common solid tumors affecting children, and it accounts for 15% of cancer-related deaths [1]. It is an embryonal malignancy of the autonomic nervous system. NB may present with a heterogeneous phenotype and clinical outcome with some tumors exhibiting (relative) good prognosis, not even requiring intervention (as in neonatally diagnosed ones), and others exhibiting an early aggressive and metastatic behavior with multiple organ dysfunction and high mortality [1]. In individuals with high-risk disease, the 5-year survival rate is less than 50%; even after radiation therapy, the loco-regional relapse is still high (50%), and these patients have a 5-year survival of only 8% [2]. The hyperactivation of oncogenic signaling pathways, such as epidermal growth factor receptor/mitogen-activated protein kinase (EGFR/MAPK), as well as the overexpression of EGF family ligands, exerts an important role in NB growth and progression [3,4,5]. Since the high expression of EGFR has been associated with enhanced tumor growth and chemoresistance in neuroblastomas, the pharmacological inhibition of EGFR is a clinical approach widely used for cancer treatment. However, some tyrosine kinase inhibitors are not effective on the MAPK pathway [6], leaving uncovered an important issue to solve. Recently, we demonstrated that lysosomal cathepsin D (CD), a ubiquitous soluble aspartic endopeptidase, contrasts neuroblastoma cell proliferation [7]. The high expression of CD reduced the sensitivity to EGF stimulation and diminished ERK 1/2 activation in human SH-SY5Y NB cells cultured in the 2D condition. Accordingly, the data retrieved from in silico transcriptome analysis showed a better prognosis and longer overall survival in NB patients with high *CTSD* (*CTSD* is the CD gene) and high *EGFR* levels [7]. NB is prone to metastasize, a process that involves the transition from adherent to a “suspended” condition where the circulating cancer cells eventually revert to the adherent phenotype at the metastatic site. Uncovering the role of EGF and CD in the growth of NB cells in adherent and suspended conditions could help us understand how NBs form metastases and lead to the discovery of new therapeutic targets. Multicellular 3D models more accurately resemble the in vivo tumor condition, and neurospheres may mimic clusters of circulating metastatic cells that disseminate in secondary sites [8].

We found different levels of CD in 2D and 3D NB culture systems. EGF stimulation downregulated CD expression in SH-SY5Y (*MYCN* not amplified) cells, but it did not induce the same effect in the other NB (*MYCN*-amplified) cell lines analyzed, IMR-32, LAN-5 and SK-N-BE(2). Neuroblastomas show a wide genetic heterogeneity, and during tumor evolution, different clones compete for survival and overtake each other [9]. To the best of our knowledge, there are no studies showing whether and how the heterogeneous expression of one single gene (particularly, *CTSD*) influences the metabolic competition and cell behavior of the clones within the tumor context. Furthermore, whether and how the expression of CD plays a role in the switch from adherent to suspended growth in NB cells remains to be elucidated. To understand the mechanistic role of CD in the competitive growth and adhesion of NB cells, we used engineered SH-SY5Y NB cells in which CD was overexpressed (Over CD clone) or knocked-down (KD-CD clone) [7], cultured either alone or in mixed proportion to mimic the intrinsic tumor heterogeneity. High expression of CD suppressed the proliferation of adherent NB cells, yet it conferred a survival and growth advantage to free-floating spheroids. Intriguingly, when the mixed Over CD and KD-CD spheroids were switched to grow on a solid substrate, mimicking the adhesion in a metastatic site, the KD-CD clone grew faster and acquired the proliferative advantage over the other. Thus, high CD expression favors the survival of floating spheroids, but it is detrimental for growth in the adherent condition. Notably, in the Sham-transfected clone, which retains the ability to modulate protein expression, the cellular level of CD increased when grown in suspension and decreased when grown in adhesion.

These findings highlight a dual role of CD in NB cell growth and suggest that this lysosomal protease is epigenetically regulated during the reversible transition for the adherent-to-suspended-to-adherent growth of metastatic clones. Consistent with this hypothesis, we found that NBs express miRNAs targeting CD that are associated with EGF signaling and with biological processes favoring cell proliferation and cell adhesion.

The present findings open the epigenetic modulation of CD expression as a valuable complementary strategy for preventing NB metastasis.

## 2. Material and Methods

### 2.1. Cell Culture and Treatments

Human neuroblastoma cell lines SH-SY5Y, IMR-32 and SK-N-BE(2) were obtained from the American Type Culture Collection (ATCC, Rockville, MD, USA, cod. CRL-2266, cod. CCL-127, cod. CRL-2271, respectively). Human neuroblastoma cell line LAN-5 was obtained from DSMZ (German Collection of Microorganisms and Cell Cultures GmbH, cod. ACC673, Braunschweig, Germany). IMR-32, LAN-5 and SK-N-BE(2) cell lines were maintained under standard conditions (37 °C, 95 *v*/*v*% air: 5 *v*/*v*% CO_2_) in RPMI-1640 medium (cod. R8758; Sigma-Aldrich Corp., St. Louis, MO, USA), supplemented with 10% heat-inactivated fetal bovine serum (FBS, cod. ECS0180L; Euroclone, Milan, Italy), 1% glutamine (cod. G7513; Sigma-Aldrich Corp.), 1% penicillin/streptomycin (PES, cod. P0781; Sigma-Aldrich Corp.), 1% non-essential amino acids (cod. M7145; Sigma-Aldrich Corp.) and 1% sodium pyruvate (cod. S8636; Sigma-Aldrich Corp.). SH-SY5Y cells were maintained in standard conditions as previously described [7]. Treatments included 20 ng/mL epidermal growth factor (EGF, cod. E5036; Sigma-Aldrich Corp.), dissolved in 10 mM acetic acid, and 100 μM Pepstatin A, an inhibitor of cathepsin D (PstA, cod. P4265; Sigma-Aldrich Corp.). SH-SY5Y stable transfectant clones Sham, knock-down CD (KD-CD) and overexpressing CD (Over CD) were generated in our laboratory [7] and have been cultivated alone or in combination for co-culture experiments both in the 2D and 3D systems, in different proportions: 50% KD-CD + 50% Over CD cells (ratio 1:1), 25% KD-CD + 75% Over CD (ratio 1:3) and 75% KD-CD + 25% Over CD cells (ratio 3:1). In the two-dimensional system (2D), cells were plated at a density of 40,000 cells/cm^2^ in Petri 60 mm, whereas for the 3D cultures, 1,000,000 cells were seeded in non-adherent Petri dishes for each experimental condition.

### 2.2. Cell Counting and Doubling Time Calculation

The cells were seeded in 12-well plates (2000–20,000–50,000 cells/cm^2^ depending on the experiment), allowed to adhere for at least 24 h and then treated with 20 ng/mL EGF and/or 100 μM PstA, as indicated. The medium was refreshed every 24 or 48 h, as indicated in figure legends. At each time point, cells were collected and counted in triplicate with Trypan blue solution. Cell counting was performed following the protocol previously described [7]. Doubling time (Dt) was calculated through the free software Doubling Time Online Calculator (v.3.1.0) (http://www.doubling-time.com/compute.php, accessed on 3 November 2023).

### 2.3. Clonogenic Assay

The cells were seeded in 6-well plates at the density of 2000 cells/well, treated with EGF and cultivated for 10 days to allow colony formation [10]. New colonies were stained with 0.5% crystal violet solution, as previously described [7]. Images of each experimental condition were acquired, and the number of colonies formed was estimated by photometric measurements with CellCounter software (v2.0.1).

### 2.4. Antibodies

The following primary antibodies were employed for Western blotting: mouse anti-β-tubulin (1:1000, cod. T5201; Sigma-Aldrich Corp.), mouse anti-β-actin (1:2000, cod. A5441; Sigma-Aldrich Corp.), rabbit anti-GAPDH (1:1000, cod. G9545, Sigma-Aldrich Corp.), mouse anti-cathepsin D (1:100, cod. IM03; Calbiochem, St. Louis, MO, USA), mouse anti-histone H3 (1:500, cod. 61475; Active Motif, Carlsbad, CA, USA). The secondary antibodies used for Western blot analysis were the following: horseradish peroxidase-conjugated goat anti-mouse IgG (1:10,000, cod. 170-6516; Bio-Rad, Hercules, CA, USA) and horseradish peroxidase-conjugated goat anti-rabbit IgG (1:10,000, cod. 170-6515: Bio-Rad, Hercules, CA, USA). The primary antibodies employed for immunofluorescence staining are listed below: mouse anti-cathepsin D (1:100, cod. IM03; Calbiochem), rabbit anti-cathepsin D (1:100; EMD Biosciences, Calbiochem, San Diego, CA, USA), rabbit anti-p27 (1:100, cod. 2552; Cell Signaling, Danvers, MA, USA), rabbit anti-Ki-67 (1:100, cod. HPA001164; Sigma-Aldrich), mouse anti-E-cadherin (1:50, cod. 14472S; Cell Signaling) and rabbit anti-N-cadherin (1:50, cod. 4061S; Cell Signaling). The secondary antibodies were goat-anti rabbit IgG Alexa Fluor Plus 488 (1:1000, cod. A32731; Invitrogen, Waltham, MA, USA) and goat-anti mouse IgG Alexa Fluor Plus 555 (1:1000, cod. A32727; Invitrogen).

### 2.5. Western Blotting

SH-SY5Y, IMR-32, LAN-5, SK-N-BE(2), SH-SY5Y Sham, KD-CD and Over CD transfectant clones were seeded at a density of 40,000 cells/cm^2^ on sterile 60 mm Petri dishes and allowed to adhere. For the experiment with SH-SY5Y Sham grown in the two-dimensional condition, cells were plated at a density of 4000 cells/cm^2^ and cultivated for 7 days. At the end, cells were collected in RIPA Buffer (0.5% deoxycholate, 1% NP-40, 0.1% sodium dodecyl sulfate in PBS solution) supplemented with protease inhibitor cocktail and phosphatase inhibitors (0.5 M sodium fluoride and 0.2 M sodium orthovanadate) and homogenized, as previously reported [7]. Protein content concentration was determined by Bradford assay and samples were denatured with 5× Leammli sample buffer at 95 °C for 10 min [7]. The bands were detected using Enhanced Chemiluminescence reagents (ECL, cod. NEL105001EA; Perkin Elmer, Waltham, MA, USA) and developed with the ChemiDoc XRS instrument (BioRad, Hercules, CA, USA). The Western blotting data were reproduced three times independently. The intensity of the bands was estimated by densitometry using Quantity One software (v.4.5) (BioRad, Hercules, CA, USA). Densitometric data represent the ratio between the intensity of the bands of the protein of interest and of the housekeeping (referred as fold increase ± S.D).

### 2.6. Immunofluorescence

SH-SY5Y transfectant clones were plated on sterile coverslips at the density of 30,000 cells/cm^2^, allowed to adhere and grown at least 24 h before treatment. The cells were treated with 20 ng/mL EGF for 72 h, where indicated. The coverslips were fixed and processed for immunofluorescence staining, as previously described [7]. After the incubation with primary antibodies, the coverslips were washed three times with 0.1% Triton-PBS and incubated for 1 h at room temperature with goat-anti rabbit IgG Alexa Fluor Plus 488 or goat-anti mouse IgG Alexa Fluor Plus 555 secondary antibodies, as appropriate. The nuclei were stained with the UV fluorescent dye DAPI (4,6-diamidino-2-phenylindole). The secondary antibodies and DAPI were dissolved in 0.1% Triton-PBS + 10% FBS. Finally, the coverslips were mounted onto glasses using SlowFade reagent (cod. S36936; Life Technologies, Paisley, UK), and the coverslips were acquired at the fluorescence microscope (Leica DMI6000, Leica Microsystems, Wetzlar, Germany). Different microscopic fields were randomly chosen, and representative pictures of the selected fields were shown.

### 2.7. RT-PCR

The total RNA was purified by TRIzol reagent (cod. T9424; Sigma Aldrich), and the total cDNA was synthesized following the standard protocol of RevertAid H Minus first strand cDNA synthesis kit (cod. K1632; Thermofisher, Waltham, MA, USA), as indicated by the purchaser. RT-PCR (35 cycles) was performed according to the manufacturer’s instructions with Recombinant Taq DNA Polymerase (cod. 10342-020; Invitrogen), starting from 1 μL of cDNA. The following primers were used for CD (forward 5′-ACCTCGTTTGACATCCACTATG-3′; reverse 5′-TGCTCAGGTAGAAGGAGAAGA-3′) and for β-Actin (forward 5′-GATCAAGATCATTGCTCCTCCTGAGCGCA-3′; reverse 5′-GTCTCAAGTCAGTGTACAGGTAAGCCCT-3′). The RT-PCR products were analyzed by 1.5% agarose gel electrophoresis.

### 2.8. 3D Spheroid Forming Assay

The 3D multicellular spheroids were cultured based on our previous work [11]. The cells were cultured in specific 35 mm Petri dishes coated with 5 mg/mL poly 2-hydroxyethyl methacrylate (poly-HEMA, cod. P3932; Sigma-Aldrich) to prevent cell adhesion. The poly-HEMA stock solution (120 mg/mL) was prepared in 95% ethanol and dissolved under rotation overnight at 50 °C. The day after, the stock solution was diluted in 95% ethanol. The petri dishes were coated with the poly-HEMA working solution (5 mg/mL) and left under the biological hood to completely dry. Then, 1,000,000 cells/Petri were seeded and maintained in culture for 7 days after treatment. Fresh medium was gently added every 48 h and supplemented with 20 ng/mL EGF and/or 100 μM PstA, as indicated. The spheroids’ growth was monitored by taking pictures with a phase contrast microscope (magnification 20×, Zeiss AXIOVERT 40 CFL, Jena, Germany) at each time-point. The area of 3D spheroids was calculated using the ImageJ software (v.1.48) and indicated as an arbitrary unit (A.U.).

In a 3D-to-2D experiment, SH-SY5Y transfectant and IMR-32, LAN-5 and SK-N-BE(2) cells were initially plated in poly-HEMA-coated Petri dishes (500,000 per 35 mm dish) and allowed to grow as neurospheres until the third day; then, the 3D cell aggregates were collected, centrifuged and reseeded in adherent Petri dishes. The adhesion and growth capacity of the transferred neurospheres were monitored for up to 72 h by imaging. The area of the secondary colonies grown on plastic was determined in several random fields using ImageJ software (v.1.48). Finally, after 72 h of culture in the adherent condition, the cells were harvested in RIPA buffer and processed for Western blot analysis.

To perform the immunofluorescence in 3D-to-2D cells, the neurospheres produced as above were collected, centrifuged and re-seeded on poly-lysine-coated coverslips, allowing them to develop secondary colonies for an additional two days. The coverslips were then fixed and stained for E- and N-cadherin immunofluorescence, as described in Section 2.6.

### 2.9. Mimicking In Vitro Tumor Heterogeneity of CD Expression

To mimic in vitro tumor heterogeneity of CD expression, a mixture of Over CD and KD-CD clones in different proportions (ratios of 1:1, 1:3 and 3:1, respectively) was cultured in 2D or in 3D conditions.

### 2.10. Statistical Analysis

Statistical analysis was performed with GraphPad Prism 6.0 software (San Diego, CA, USA). Bonferroni’s multiple comparison tests after one-way or two-way ANOVA analysis (unpaired, two-tailed) or unpaired *t*-test analysis were employed as reported in detail in each Figure legend. The significance was considered as follows: **** *p* <0.0001; *** *p* < 0.001; ** *p* < 0.01; * *p* < 0.05. The data are reported as average ± S.D.

### 2.11. Bioinformatic Analysis

Kaplan–Meier curves, correlation studies, oncoprint and biological processes were obtained by extracting clinical data from the TCGA database (www.portal.gdc.cancer.gov/, last accessed on 22 February 2024). RNA-seq and the corresponding clinical data (including overall survival status, INSS stage and mRNA expression) from pediatric neuroblastoma patients (TARGET 2018, comprising 248 patients after filtering out datasets with insufficient survival information) were downloaded from cBioportal.org. The patients were grouped based on the level of mRNA expression. Low versus high groups were defined relative to the median expression level of the overall patient cohort. The correlation between the mRNA expression of the relevant biomarker, *MYCN*, *CTSD* and the international neuroblastoma staging system (INSS) stage are represented in histograms. Pearson’s and Spearman’s correlation analyses were performed to identify the genes correlated with *MYCN* and identify the genes and miRNAs correlated with *CTSD*. Scatter plots were employed to represent the correlation between the expression of relevant biomarkers in the patient cohort. Regression was estimated by calculating Pearson’s correlation coefficients (r) and the relative *p*-values.

TBtools (https://github.com/CJ-Chen/TBtools/; (accessed on 25 February 2024) was used to identify differentially expressed genes (DEGs) and differentially expressed (DE) miRNAs in correlation with *CTSD* expression. To identify the DEGs, the cut-off criteria were set based on Spearman’s correlation values. Specifically, the correlation coefficient value was greater than +0.45 for positively correlated and lower than −0.45 for negatively correlated; a *p*-value < 0.0001 (−log10 (*p*-value)) threshold was fixed above 5.0. The cut-off criteria were set based on Spearman’s correlation values to identify the DE miRNAs. In DEGs, the correlation coefficient value was greater than +0.25 for positively correlated and lower than −0.25 for negatively correlated; a *p*-value < 0.001 (−log10 (*p*-value)) threshold was fixed above 5.0. The correlation analyses were represented as Volcano plots.

The DAVID bioinformatic functional annotation tool (https://david.ncifcrf.gov/summary.jsp; accessed on 20 February 2024)) was used to analyze Gene Ontology (GO) biological processes, and Kyoto Encyclopedia of Genes and Genomes (KEGG) pathways were obtained with the help of negative DEGs. The data are presented in bar graphs displaying the number of transcripts for each negatively associated biological process.

DIANA Tools (diana.imis.athena-innovation.gr/; accessed on 15 February 2024) was used to retrieve predicted *CTSD* negatively correlated miRNAs and GO processes in which they are involved. DIANA-mirPath (v.3.0) was applied to obtain miRNA- and pathway-related information for these analyses. mirPath utilizes predicted miRNA targets (in CDS or 3′UTR regions) provided by the DIANA algorithms (TarBase v.7.0) or even experimentally validated miRNA interactions.

The statistical analyses were performed using R (v.4.3.2, The R Foundation for Statistical Computing, Vienna, Austria) and SAS software (v.9.4, SAS Institute Inc., Cary, NC, USA). The log-rank test was used to determine the statistical significance. The *p*-value ≤ 0.05 was considered as significant.

## 3. Results

### 3.1. The Prognostic Value of MYCN and CTSD and Their Correlation with EGF Signaling in Pediatric Neuroblastoma Patients

**Figure 1:**
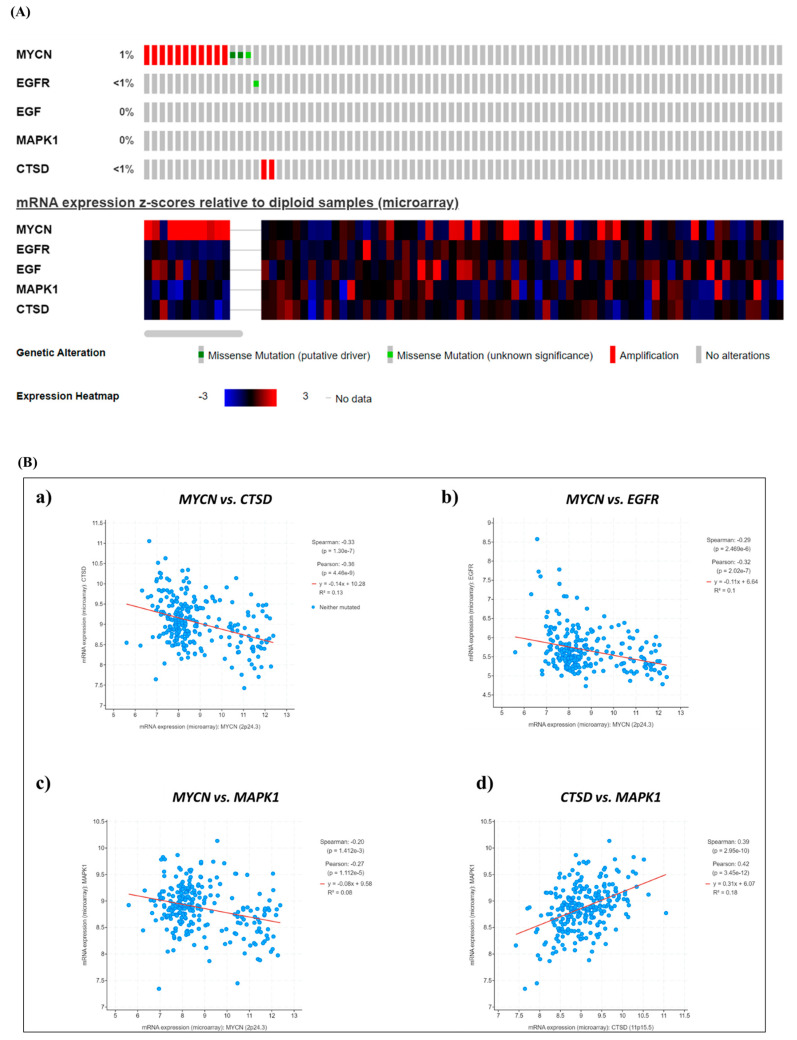
Neuroblastoma patients bearing a tumor with *MYCN* amplification or high *MYCN* mRNA levels show a poor prognosis. (**A**) Oncoprint of somatic mutations shows the genetic alterations and mRNA expression levels of *MYCN*, *EGFR*, *EGF*, *MAPK1* and *CTSD* in pediatric NB patients (TARGET, 2018). (**B**) Scatter plots representing the correlations between *MYCN*–*CTSD* (**a**), *MYCN–EGFR* (**b**), *MYCN*–*MAPK1* (**c**) and *CTSD–MAPK1* (**d**) mRNA expression.

In our previous work, we found that prognosis was improved in patients bearing a neuroblastoma with high expression of *CTSD* regardless of the expression of *EGFR* [7]. Here, we looked for the correlation between the *MYCN*, *CTSD*, *EGFR* and *MAPK1* genes and checked the prognostic value of *CTSD* in the context of *MYCN* positive and negative neuroblastomas. First, we searched for the pediatric neuroblastoma patients (TARGET 2018) available in the TCGA portal to monitor the mutation profiles and mRNA expression of *MYCN*, *EGFR*, *EGF*, *MAPK1* and *CTSD.* The oncoprint (Figure 1A, upper part) shows that out of 248 pediatric neuroblastoma patients, *MYCN* was amplified in 11 patients, and a missense mutation was found in 3 patients (1%). *EGFR* and *CTSD* genes were altered in less than 1% of the cohort, whereas *EGF* and *MAPK1* were unaffected by gene mutation or chromosomal alteration. Additionally, the heatmap depicting the mRNA expression (Figure 1A, bottom part) indicates that most neuroblastoma patients display high levels of *MYCN*, while the mRNA levels of *EGFR*, *EGF*, *MAPK1* and *CTSD* are low or very low. A correlation analyses revealed that the expression of *CTSD, EGFR* and *MAPK1* was inversely correlated with that of *MYCN* (Figure 1B, panels a–c), whereas *CTSD* expression was positively correlated with *MAPK1* (Figure 1B, panel d).

**Figure 2:**
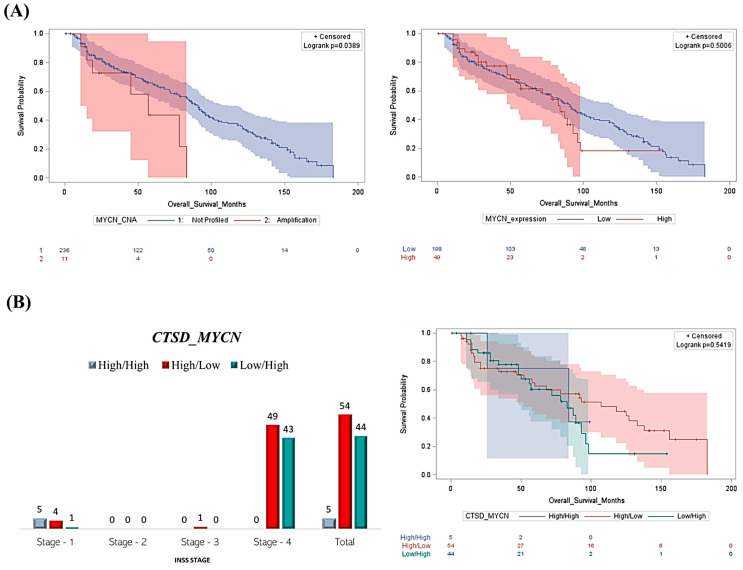
Patients with high *CTSD* and low *MYCN* expression show better clinical outcomes despite the advanced stage. (**A**) The Kaplan–Meier curves reporting the overall survival rate were obtained based on *MYCN* copy number alterations and *MYCN* mRNA expression (high versus low). (**B**) Graph reporting the INSS staging of NB patients based on *CTSD* and *MYCN* expression and Kaplan–Meier plot representing the overall survival of NB patients according to a differential expression of *CTSD* and *MYCN* (High/High, High/Low, and Low/High groups, respectively).

Next, we focused on the clinical significance of such correlations. As predicted, patients with *MYCN* amplification or high mRNA expression displayed the worst prognosis compared to the unaltered or low-expressor patients (Figure 2A). Next, we compared the prognosis of the patients grouped based on combined mRNA expression of *CTSD* and *MYCN* as High/High (H/H), High/Low (H/L) and Low/High (L/H), respectively. Intriguingly, very few patients had a H/H profile and were diagnosed at stage 1. The majority of patients had a H/L or L/H profile and were diagnosed at stage 4 (Figure 2B). Furthermore, the Kaplan–Meier overall survival curves indicated that patients displaying high *CTSD* and low *MYCN* (H/L profile) mRNA expression had a better clinical outcome compared to those bearing a tumor with low *CTSD* and high *MYCN* (L/H profile) expression (Figure 2B). These data suggest that *CTSD* expression is a determinant prognostic factor in NB patients.

### 3.2. EGF Reduces Cathepsin D Levels in MYCN-Not-Amplified SH-SY5Y Cells

**Figure 3:**
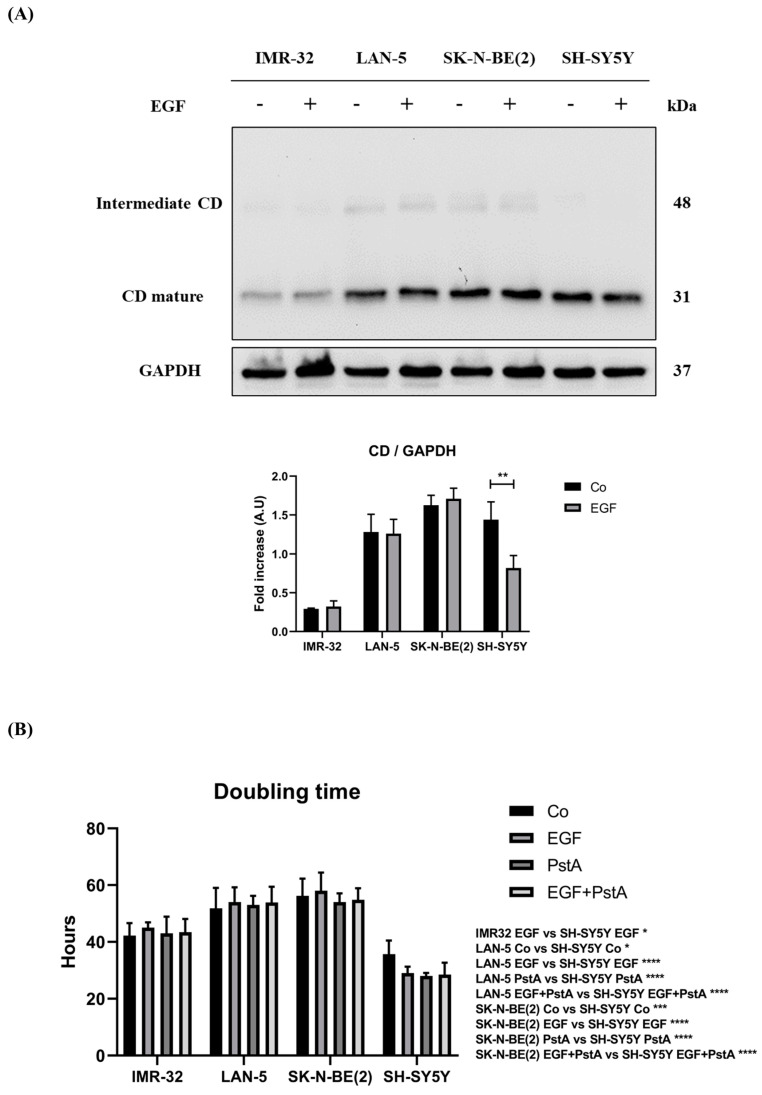
EGF differentially modulates cathepsin D levels and cell proliferation in *MYCN*-amplified and *MYCN*-not-amplified neuroblastoma cell lines. IMR-32, LAN-5, SK-N-BE(2) and SH-SY5Y cells were cultured for 72 h, medium was renewed, and EGF was re-added every day. Samples were collected after 72 h and processed for Western blot analysis of CD. (**A**) Western blotting of CD expression in NB cell homogenates. The membrane was probed with GAPDH as loading control. The blot is representative of three independent experiments. Densitometry of the bands is reported in the histogram. One-way ANOVA test was performed. Significance was considered as follow: ** *p* < 0.01. (**B**) Cells were seeded, allowed to adhere for 24 h and then counted before (time 0) and at the end of the treatment (72 h) with PstA, EGF or both. Doubling time (Dt) was calculated as described in the methods. Two-way ANOVA test was performed. Significance was considered as follow: **** *p* < 0.0001; *** *p* < 0.001; * *p* < 0.05. “Co” refers to untreated cells (control).

Our previous work showed an EGF-induced reduction in CD levels in *MYCN*-not-amplified SH-SY5Y neuroblastoma (NB) cells [7]. Here, we compared CD expression in this latter cell line with that of *MYCN*-amplified IMR-32, LAN-5 and SK-N-BE(2) NB cells, in the presence or absence of EGF. IMR-32 showed the lowest, while SK-N-BE(2) the highest, basal expression of CD. Moreover, we observed that EGF stimulation did not affect CD levels in IMR-32, LAN-5 and SK-N-BE(2). In line with our previous findings, we found a significant reduction (around 50%) in cathepsin D expression in EGF-treated SH-SY5Y cells (Figure 3A). To determine the influence of CD activity on the proliferative potential, we calculated the doubling time (Dt) of these NB cell lines treated with EGF in the presence/absence of Pepstatin A (PstA), a specific inhibitor of CD (Figure 3B). In the co-treatment condition, PstA was added before EGF administration to inhibit the enzymatic activity of CD. IMR-32, LAN-5 and SK-N-BE(2) displayed a Dt of 42, 52 and 56 h, respectively, which was not affected by the addition of EGF and PstA. On the other hand, SH-SY5Y showed a doubling time (Dt) of 36 h, which was reduced to 29 h by EGF, to 28 h by PstA and to 28.5 h by EGF + PstA, indicating that the effects of EGF and of PstA were similar and not synergistic nor additive.

### 3.3. SH-SY5Y Cells Grown as 2D or 3D Express Different Level of Cathepsin D

**Figure 4:**
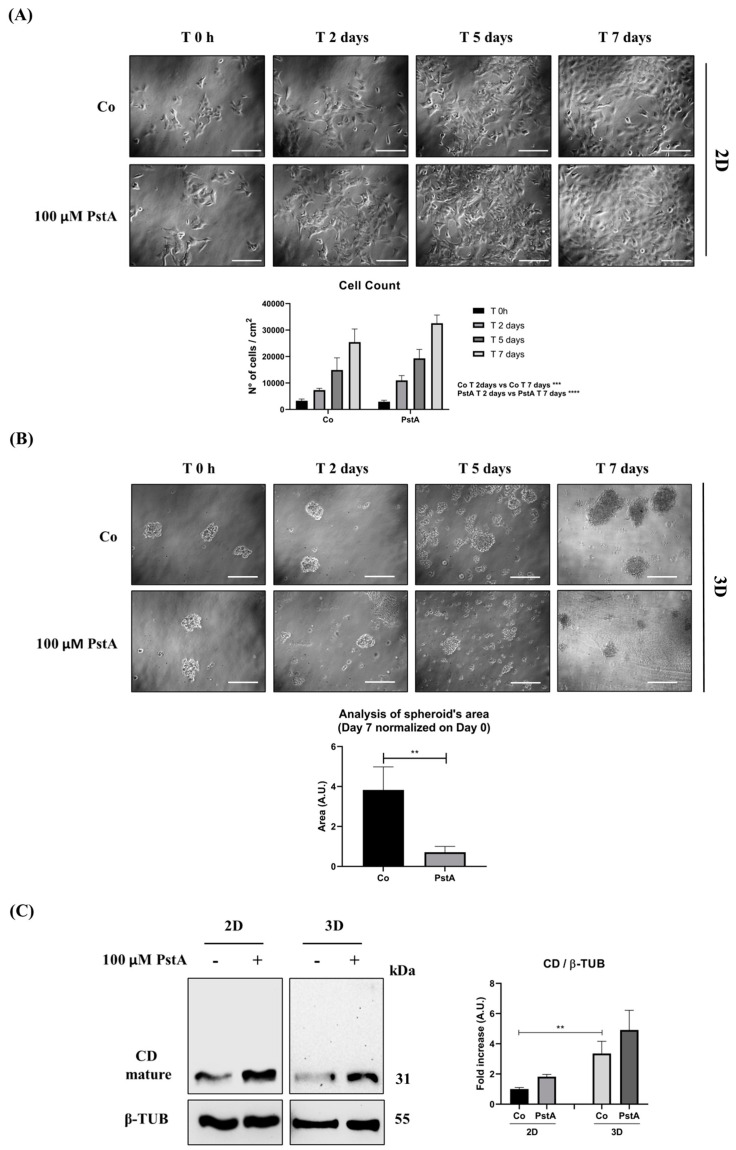
Cathepsin D expression differs in 2D and 3D SH-SY5Y cell cultures. SH-SY5Y cells were seeded both in adherent and non-adherent Petri dishes, at 4000 cells/cm^2^ and 1,000,000 cells/Petri, respectively, and allowed to grow for 48 h. Medium was refreshed every 48 h, supplemented with 100 μM PstA where indicated. (**A**) Cell growth was monitored at the phase-contrast microscope and images were acquired at the time point indicated (time 0 h, 2-, 5- and 7-days). Scale bar = 100 μm; magnification = 20×. Viable SH-SY5Y cells grown in adherent condition were counted, and data from triplicate for each experimental condition are shown in the graph. One-way ANOVA test was performed. Significance was considered as follows: **** *p* < 0.0001; *** *p* < 0.001. (**B**) The spheroid’s growth was monitored at the phase-contrast microscope, and images were acquired at the time point indicated (time 0 h, 2-, 5- and 7-days). Scale bar = 100 μm; magnification = 20×. The quantification of 3D spheroid’s size was performed with ImageJ software (v.1.48). The area is indicated as arbitrary unit (A.U.). Data represent the average ± S.D. calculated for at least 5 to 10 spheroids for each condition in three separate experiments. *t*-test was performed. Significance was considered as follows: ** *p* < 0.01. (**C**) Western blotting analysis of CD expression in 2D and 3D cell homogenates. The membrane was probed with β-tubulin as loading control. The blot is representative of three independent experiments. Densitometry of the bands is reported in the histogram. Two-way ANOVA test was performed. Significance was considered as follow: ** *p* < 0.01. “Co” refers to untreated cells (control).

The above data indicate that *MYCN*-amplified NB cells are not responsive to EGF, while SH-SY5Y cells (*MYCN*-not-amplified) proliferate at a higher rate, and yet, their proliferation rate increases (the Dt was reduced by 25%) when CD is reduced by EGF or inhibited by PstA We investigated further the impact of CD on the growth of *MYCN*-not-amplified SH-SY5Y NB cells grown in adherent (2D) and non-adherent (3D) conditions. When grown in non-adherent condition, the cells form multicellular aggregates with a spherical-like shape in which the cellular behavior is different from that in the 2D system. In fact, it has been shown that 3D spheroids of diameter >300–500 µm present with proliferating cells in the most peripheral layer, while the intermediate layer contains quiescent cells, and the most inner core is necrotic [12]. We employed PstA to determine the contribution of CD proteolytic activity in the anchorage-dependent and anchorage-independent growth of SH-SY5Y cells (Figure 4A,B). The enzymatic inhibition of CD increased the growth (cell number) of adherent SH-SY5Y cells by some 57%, 33% and 26% at day 2, 5 and 7, respectively (Figure 4A). In contrast, in 3D cultures, CD inhibition inhibited the growth of multicellular spheroids, whose size was 4-times smaller than that of untreated neurospheres with active CD (Figure 4B). These data suggest that in the absence of adhesion CD confers survival and growth advantage, which is nullified when its proteolytic activity is inhibited by PstA. By Western blotting, it was shown that SH-SY5Y cells grown as 3D spheroids expressed approx. 5-times more CD than the counterpart grown as 2D (Figure 4C). To be noted, PstA slightly (not significantly) favored the accumulation of mature CD in the lysosomes (Figure 4C). From these data, we conclude that CD is differently modulated in SH-SY5Y cells cultured in anchorage-dependent and anchorage-independent conditions, and its activity is necessary for the growth in the latter condition.

### 3.4. Cathepsin D Expression Differentially Affects the 2D and 3D Growth of SH-SY5Y Cells

**Figure 5:**
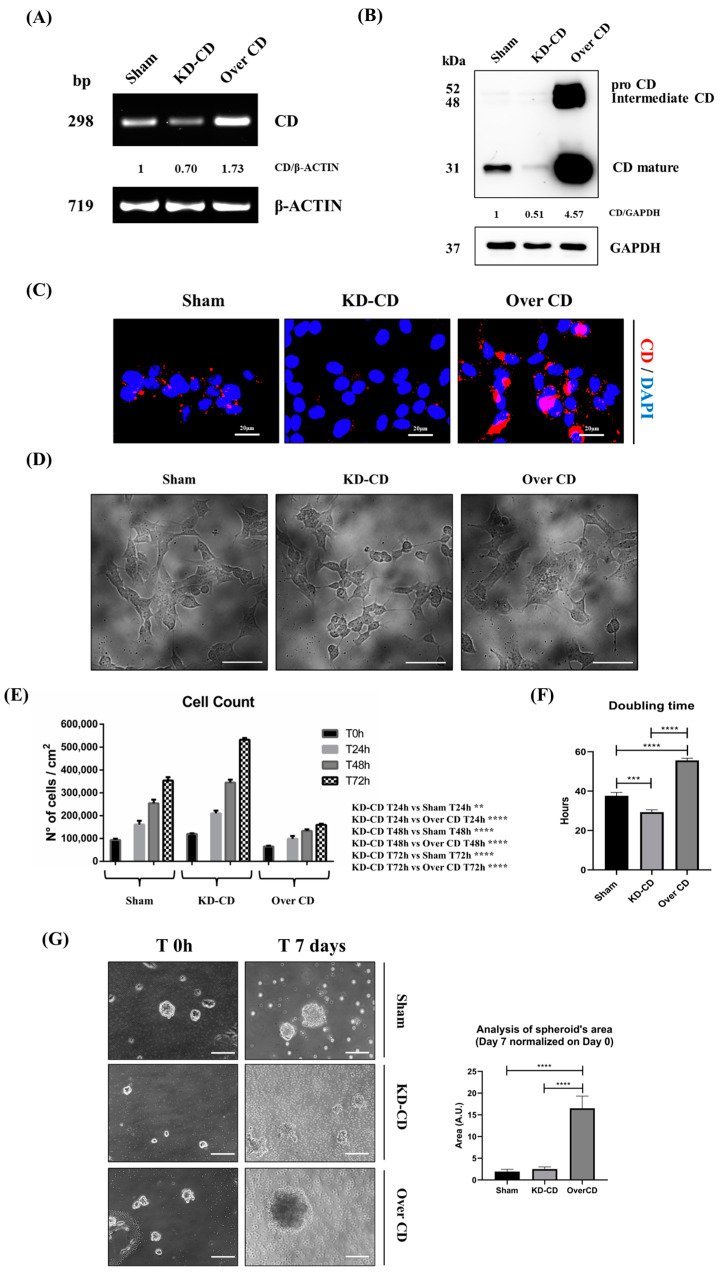
SH-SY5Y Sham, KD-CD and Over CD clones show different growth rates. (**A**) Agarose gel electrophoresis of the products of the RT-PCR for CD and β-actin. Densitometric analysis of CD/actin ratio is shown. (**B**) Western blotting showing the expression of CD in the three SH-SY5Y clones. The blot was probed with GAPDH as loading control. Densitometric analysis of CD/GAPDH ratio is shown. (**C**) Immunofluorescence performed on SH-SY5Y clones. Cells were seeded on sterile coverslips and then fixed and stained for CD (red); nuclei were marked with the UV fluorescent dye DAPI. Scale bar = 20 µm; magnification = 63×. (**D**) Phase contrast images of the three SH-SY5Y clones. (**E**) Graph representing cell count. Cell counting was performed in triplicate for each experimental condition. Two-way ANOVA test was performed. Significance was considered as follows: **** *p* < 0.0001; ** *p* < 0.01. (**F**) Graph reporting the doubling time of the three SH-SY5Y clones. One-way ANOVA test was performed. Significance was considered as follows: **** *p* < 0.0001; *** *p* < 0.001. (**G**) 3D cultures of neuroblastoma clones. SH-SY5Y Sham, KD-CD and Over CD cells were seeded on non-adherent Petri dishes and maintained in culture for 7 days. New fresh medium was replaced every 48 h. The 3D spheroid’s growth was monitored at the phase-contrast microscope, and pictures were acquired. Scale bar = 100 μm; magnification = 20×. The quantification of 3D spheroid’s size was performed with ImageJ software (v.1.48). The area was indicated as arbitrary unit (A.U.). Data represent the average ± S.D, calculated for at least 5 to 10 spheroids for each condition in three separate experiments. One-way ANOVA test was performed. Significance was considered as follows: **** *p* < 0.0001.

To better assess the role of CD in the anchorage-dependent and anchorage-independent growth of neuroblastoma, we took advantage of human neuroblastoma SH-SY5Y stable transfectant clones engineered in our laboratory that are silenced (knocked-down, KD-CD) or overexpressed (Over CD) for this protease [7]. The effectiveness of such genetic manipulations is here confirmed by RT-PCR (Figure 5A), Western blotting (Figure 5B) and immunofluorescence staining of CD (Figure 5C). CD is synthesized as a glycosylated inactive precursor (proCD of 52 kDa) in transit in the Golgi apparatus that is subsequently transported to the endosome where it is processed to an active intermediate single-chain form (ICD, 48 kDa) that eventually matures within the lysosome as the most active double-chain form, made up of a large chain (LC, 31 kDa) and a small chain (SC, 13 kDa) [13]. It appears obvious that CD is highly expressed in the Over CD clone, while it is barely detectable in the KD-CD clone. SH-SY5Y culture presents a mixture of two cell types that differ on morphological and biological bases: the “N-type”, showing a neuroblastic-like morphology and aggressive behavior, and the “S-type”, showing a mesenchymal-like (flattened) morphology and less malignant behavior [14]. The N-type shows a strong cell-to-cell adhesion and forms aggregates at high cellularity, while the S-type tends to adhere to the substrate and its growth is contact-inhibited [14]. Upon microscopic inspection, it appears that the N-type is prevalent (approx. 80%) in the KD-CD clone, while the S-type is prevalent (approx. 70%) in the Over CD clone (Figure 5D).

In the 2D culture, the KD-CD clone displayed the highest, while the Over CD clone displayed the lowest proliferation rate, compared to the Sham-transfected clone (Figure 5E). Accordingly, the Dt of Over CD clone was approximately 50% increased (from 38 h to 56 h) while the Dt of KD-CD was approximately 25% reduced (from 38 h to 29 h) compared to that reported for the Sham-transfected counterpart (Figure 5F). Strikingly, the Dt of KD-CD is very close to that of the wild-type counterpart cultivated in the presence of EGF or of PstA (compare Figure 3B and Figure 5F).

Then, we assayed the growth rate of these clones cultured as 3D spheroids. Compared to time zero, after 7 days, the dimension (area) of the spheroids doubled in the case of the sham clone, and increased 2.5- and 15-times in the cases of the KD-CD and Over CD clones, respectively (Figure 5G).

### 3.5. Cathepsin D Overexpression Contrasts While Cathepsin D Silencing Enhances EGF-Induced Clonogenic Growth in 2D System

**Figure 6:**
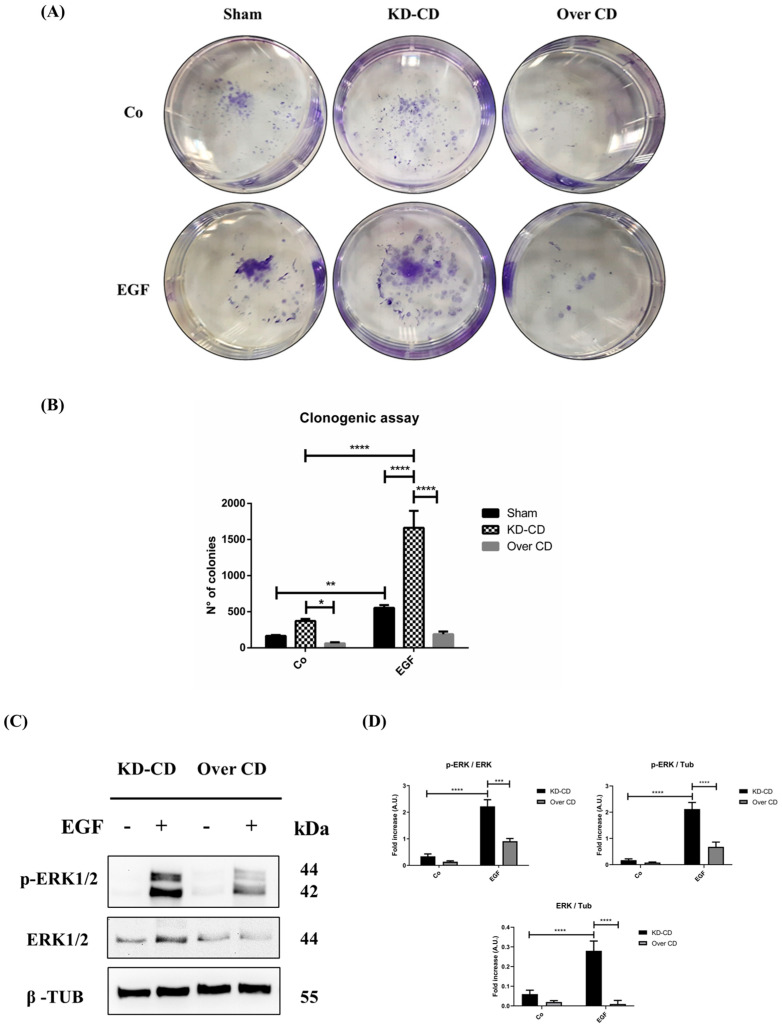
EGF stimulation of neuroblastoma growth depends on cellular level of cathepsin D. (**A**) Clonogenic assay performed on SH-SY5Y transgenic clones upon stimulation with 20 ng/mL EGF. Colonies were stained as described in the Methods section. (**B**) Cell growth and number of colonies were estimated through photometric measurements and CellCounter Software and are shown in the graph. Data ± S.D. are representative of three independent replicates. (**C**) Western blotting showing the phosphorylation of ERK 1/2 (p-ERK 1/2) in KD-CD and Over CD cells treated with EGF. Cells were cultured for 72 h; medium was renewed and EGF re-added every day. Samples were processed for Western blot analysis of p-ERK and total ERK. Membranes were probed with β-tubulin as loading control. All blots are representative of three independent experiments. (**D**) Densitometry of the p-ERK/ERK, p-ERK/Tubulin and ERK/Tubulin ratios are reported in the histograms. Two-way ANOVA test was performed in all the statistical analysis reported in the figure. Significance was considered as follows: **** *p* < 0.0001; *** *p* < 0.001; ** *p* < 0.01; * *p* < 0.05. “Co” refers to untreated cells (control).

Clonogenic assay confirmed that transgenic SH-SY5Y KD-CD cells had higher proliferation rates, while those overexpressing CD had lower proliferation rates compared to their Sham-transfected counterpart (Figure 6A,B). EGFR stimulation exacerbates the growth and aggressive behavior of NBs [4,5]. The administration of 20 ng/mL EGF, which is in the range of cell growth stimulation [15], markedly increased the colony-forming ability of CD knockdown cells (Figure 6A). When stimulated with EGF, the colony formation increased differently in the three clones, with an increment of 3.4-folds, of 4.5-folds, and of 3-folds for Sham-transfected, KD-CD and Over CD, respectively (Figure 6A,B). Particularly, the number of newly formed colonies in EGF-treated KD-CD clones was 3- and 8.8-times higher compared to Sham and Over CD, respectively, especially in presence of EGF. To substantiate this effect, we assayed the activation of the cell proliferation pathway downstream to EGFR. KD-CD cells, which are highly proliferative, show basally higher levels of ERK 1/2 and its phosphorylated form than Over CD cells (Figure 6C,D). Upon exposure to EGF, phosphorylation of ERK 1/2 increased in both the cell type though the absolute level (versus Tubulin) was 2-fold higher in KD-CD cells compared to Over CD cells (Figure 6C,D). These data definitively confirm that in adherent 2D conditions, low or null expression of CD favors NB cell growth. Furthermore, these data indicate that KD-CD cells are more prone to respond to EGF-induced growth stimulation.

### 3.6. Knocked-Down CD Clone Overtakes over CD Clone in EGF-Stimulated Growth of Mixed Cultures in 2D System

**Figure 7:**
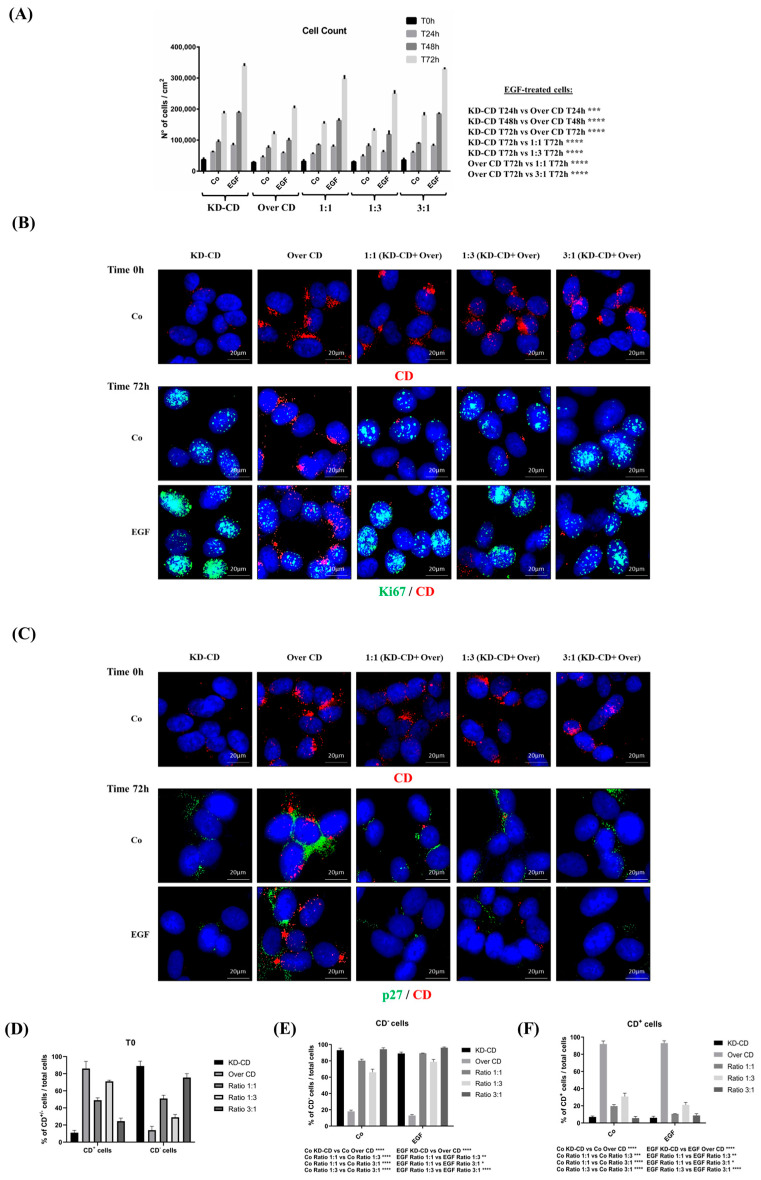
CD knockdown clone expresses high level of nuclear Ki-67 proliferation marker and reduced p27 cell cycle inhibitor. (**A**) Cell counting of viable cells of KD-CD or Over CD or a mix of both at the ratio indicated, in the absence or the presence of EGF. Medium was renewed and EGF added every 24 h. The cells were cultivated for 24, 48 and 72 h. (**B**,**C**) At the beginning of the experiment (time 0), cells were fixed and stained for CD to confirm the composition of co-cultures. Immunofluorescence double staining of CD and Ki-67 or p27 was performed on SH-SY5Y KD-CD, Over CD and mixed cultures at different ratios: 50% KD-CD + 50% Over CD cells (1:1), 25% KD-CD + 75% Over CD (1:3) and 75% KD-CD + 25% Over CD cells (3:1). Fresh medium was replaced every day, and EGF was added as indicated. After 72 h of treatment, cells were fixed and stained for Ki-67 (green)/CD (red) (**B**) and p27 (green)/CD (red) (**C**). Scale bar = 20 µm; magnification = 63×. Representative images of three independent experiments are shown. (**D**–**F**) Percentages of CD negative and CD positive cells relative to the total cell population, calculated in random fields, are shown in the graphs. Two-way ANOVA test was performed in all the statistical analysis reported in the graphs. Significance was considered as follows: **** *p* < 0.0001; *** *p* < 0.001; ** *p* < 0.01; * *p* < 0.05. “Co” refers to untreated cells (control).

Tumor evolution is characterized by the presence of multiple cancer clones with different genetic backgrounds, and these clones compete for survival and overtake each other. We hypothesized that during malignant progression, NB could develop subclones expressing different levels of CD, which then would respond differently to EGF. To mimic such tumor heterogeneity, we have mixed the clones overexpressing or silenced for CD at different ratios and tested which one would take advantage to grow over the other in the absence or in the presence of EGF stimulation. In pure 2D cultures, EGF greatly stimulated the growth of KD-CD and, to a much lesser extent, that of Over CD, as expected (Figure 7A). Then, the KD-CD and Over CD clones were mixed in different proportions, at 1:1 or 1:3 or 3:1 ratio. Upon EGF stimulation, the co-culture with the highest proportion of KD-CD cells showed the highest proliferation rate (Figure 7A). Immunofluorescence staining of Ki-67, a proliferative nuclear marker, and of p27^Kip1^, a cyclin-dependent kinase inhibitor that prevents entering the cell cycle, corroborated these findings. To distinguish the two populations of KD-CD and Over CD in the mixed cultures, we co-stained the cells with an anti-CD antibody. In pure cultures, EGF strongly increased the expression of nuclear Ki-67 and decreased that of p27 in KD-CD cells, and conversely high levels of p27 and low levels of Ki-67 were observed in Over CD cells (Figure 7B,C). In co-cultures, especially in the 1:1 and 3:1 ratio of KD-CD versus Over CD, we observed high expression of Ki-67 and decreased expression of p27 in KD-CD cells, which were the most represented ones, indicating that these cells took advantage to grow over the other. The high percentage of CD-silenced cells on the total number of Ki-67^+^ cells, in both EGF-treated and untreated conditions, essentially confirmed the prevalence of the KD-CD clone in the mixed co-cultures (Supplementary Figure S1). The percentages of Ki-67^+^ and p27^+^ cells relative to the total cell population corroborated the above findings (Supplementary Figure S1).

**Figure 8:**
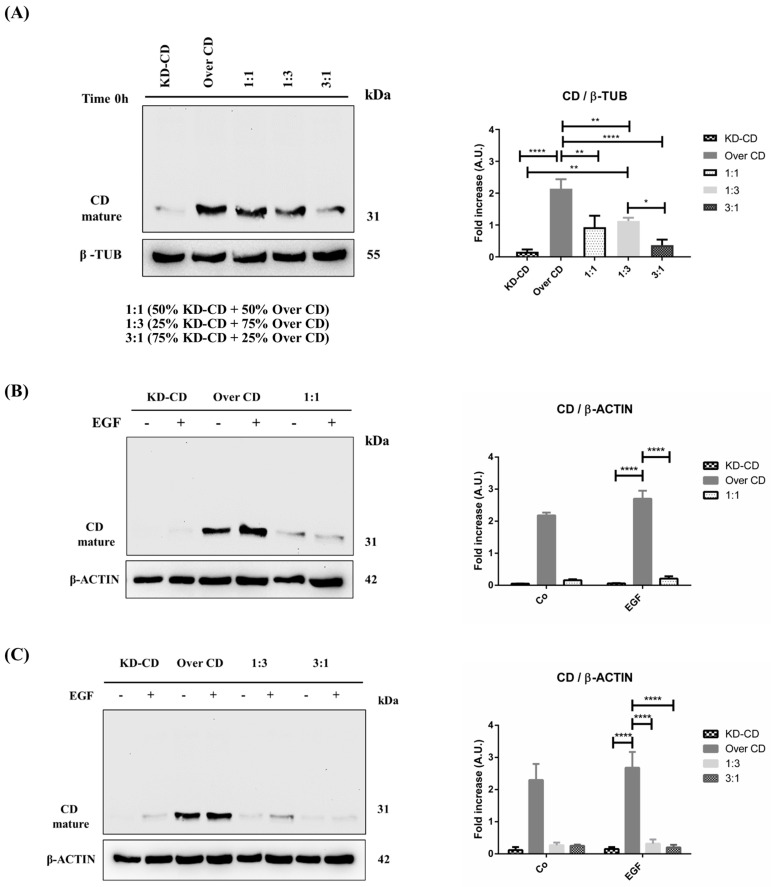
Analysis of cathepsin D content in SH-SY5Y mixed clones. Western blotting showing the expression of cathepsin D in pure cultures (KD-CD and Over CD) and in mixed co-cultures: 50% KD-CD + 50% Over CD cells (1:1), 25% KD-CD + 75% Over CD (1:3) and 75% KD-CD + 25% Over CD cells (3:1). (**A**) Samples were collected at time 0h, before EGF treatment, and processed for Western blot analysis of CD expression. One-way ANOVA test was performed. (**B**,**C**) Cells were cultured for 72 h, medium was renewed, and EGF re-added every day. Samples were collected after 72 h and processed for Western blot analysis of CD. Membranes were probed with β-actin as loading control. All blots are representative of three independent experiments. Densitometry of the bands is reported in the histograms. Two-way ANOVA test was performed. Significance was considered as follows: **** *p* < 0.0001; ** *p* < 0.01; * *p* < 0.05. “Co” refers to untreated cells (control).

We further assessed the level of cellular CD expressed in the mixed clones, first at time zero (0 h) (Figure 8A), which reflects the CD levels according to the starting proportion of the clones, and after 72 h of culture in the presence/absence of EGF (Figure 8B,C). The Over CD clone expressed approximately 2.5-folds more CD than the KD-CD clone, in which CD was barely expressed. In the absence of EGF stimulation, after 72 h, the mixed culture of the two clones at 1:1, 1:3 and 3:1 (KD-CD versus Over CD) ratios demonstrated a progressive reduction in the total content of CD. However, at 1:1 ratio the total content of CD was reduced by some 30% and not by 50%, as one would expect. Furthermore, comparing the two opposite proportion of the clones (1:3 versus 3:1) it appears that the reduction of CD in the whole homogenate is not reflecting the proportion of the clones (Figure 8A). Together with the data in Figure 7 and in agreement with our previous findings [7], the possible explanation is that in the mixed co-cultures, the Over CD cells are viable though in a resting phase, while the KD-CD cells are proliferating (and, of course, these cells do not contribute to the CD content in the whole homogenate). This implies that with time, the latter clone would overtake the former. To accelerate this process, we exposed the clones to EGF. We previously reported [7] that KD-CD cells respond to the growth-promoting effect of EGF. Consistently, EGF greatly stimulated the growth of KD-CD cells, which surpassed CD-overexpressing cells in the cultures mixed at any ratio (Figure 8B,C). It is worth noting that this effect is evident at the 1:1 ratio and, even more strikingly, at the 1:3 ratio, in which the number of Over CD cells seeded at time zero was 3-times more than that of the KD-CD cells. Since, in these transgenic clones, only endogenous CD can be modulated by EGF, it is obvious that the changes displayed in the Western blotting are largely due to changes in the proportion of the cells expressing or silenced for this protein.

### 3.7. Cathepsin D Overexpression Increases the Survival of NB Spheroids Cultivated in Suspension

**Figure 9:**
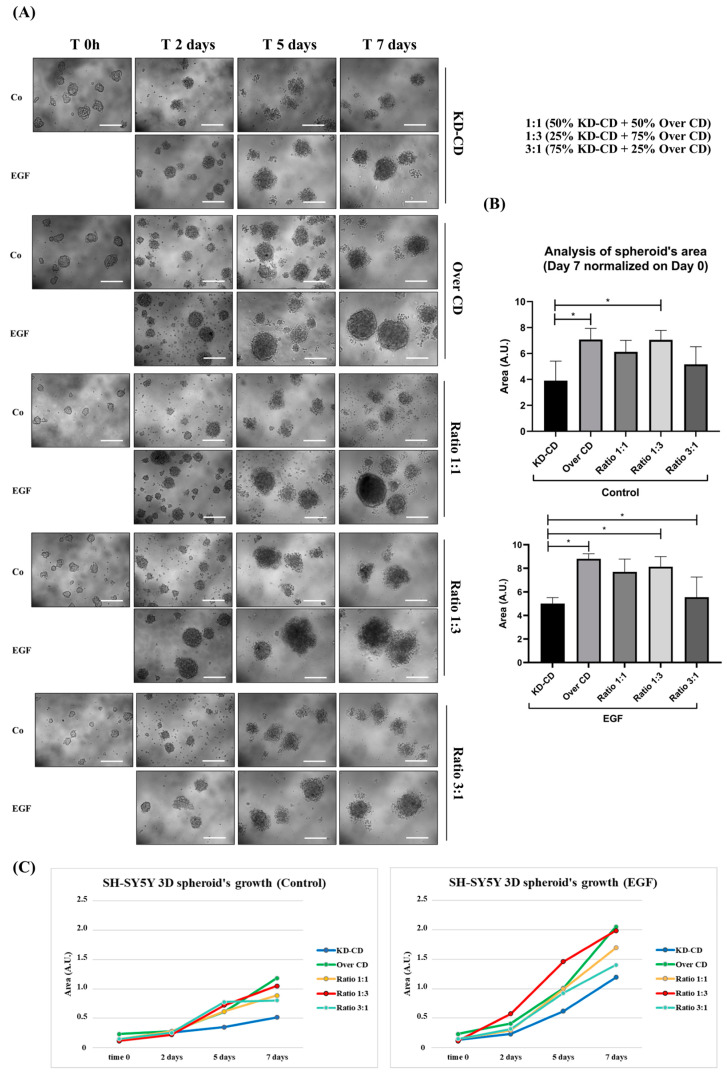
Monitoring of spheroid’s formation in pure and mixed clones co-cultured in the absence or presence of EGF. SH-SY5Y KD-CD, Over CD and co-cultures (50% KD-CD + 50% Over CD cells (1:1), 25% KD-CD + 75% Over CD (1:3) and 75% KD-CD + 25% Over CD cells (3:1)) were plated on non-adherent Petri dishes and allowed to grow for 48 h to allow spheroid formation. Cells were cultured for 7 days after the first treatment. At time 0h, cells were incubated with EGF and re-treated in fresh medium at days 2 and 5 until the endpoint of 7 days. (**A**) The 3D spheroid’s growth was monitored at the phase-contrast microscope and images were acquired at different time points (time 0 h, 2-, 5- and 7-days). Scale bar = 100 μm; magnification = 20×. (**B**) Quantification of spheroid’s size was obtained through ImageJ software (v.1.48). The area is indicated as arbitrary unit (A.U.). Data represent the average ± S.D., calculated for at least 5 to 10 spheroids for each condition in three separate experiments. Graphs show the statistically significant differences in spheroid area detected at time day 7 (endpoint) normalized at time 0 h. One-way ANOVA test was performed. Significance was considered as follows: * *p* < 0.05. (**C**) Graphs representing the increasing growth of spheroids in control and EGF-treated conditions. The area is indicated as arbitrary unit (A.U.).

Neurospheres can be assumed to be clusters of cells representing metastatic clones at the step of detachment from the primary tumor and, possibly, circulating in body fluids. At this point, it was necessary to determine the role of CD in the EGF-induced proliferation of the KD-CD and Over CD clones cultivated in suspension. To mimic tumor heterogeneity for CD expression, we co-cultured the two clones (KD-CD and Over CD) in different proportions, as detailed above. Representative images and quantification of the spheroid’s growth are shown in Figure 9A,B. The dimension of the neurospheres was larger (approximately 1.7-folds by 7 days) in Over CD than in KD-CD pure cultures (Figure 9A,B). A similar trend was observed in mixed clones at the ratios 1:1 and 1:3, but not at the ratio 3:1 in which the Over CD cells are less represented, suggesting that Over CD could take over when the mixed clones were grown in suspension. It is to be noted that the spheroid’s area data calculated at day 7 are normalized versus the area at day zero, which corresponds to 48 h from plating, and during this period, the Over CD clone already gained a proliferation advantage over the KD-CD clone (this can be appreciated in the growth curves, Figure 9C. The treatment with EGF stimulated the growth of spheroids, the effect being more prominent on the KD-CD clone, as expected. The time-dependent growth of the spheroids is shown in Figure 9C. After 7 days of EGF stimulation, the KD-CD spheroid’s size was increased by 2.4-folds while that of Over CD was increased by 1.7-folds (Figure 9C). This suggests that Over CD cells growing in suspension already have upregulated proliferative signaling, and EGF may give limited further stimulation.

We extended the study to *MYCN*-amplified NB cells (IMR-32, LAN-5 and SK-N-BE(2) cultured in a 3D non-adherent condition. Remarkably, the low CD expressing IMR-32 cell line showed the least growth rate. Additionally, as previously seen for 2D cultures, the addition of EGF and/or PstA (up to 7 days) did not significantly modulate their spheroid size, further indicating that *MYCN*-amplified NB cells are not responsive to EGF and CD inhibition (Supplementary Figure S2).

To have a more objective measure of the spheroids, in terms of cell number, we assayed the expression of histone H3 in an equal volume of cell homogenate for each condition. The H3 protein is one of the main histones composing chromatin structure and can be assumed to be an indirect readout of the cell number. The Western blotting (Supplementary Figure S3) shows high levels of histone H3 in pure Over CD culture and in the mixed cultures at the ratio 1:1 and 1:3, particularly in EGF condition. In EGF-treated pure cultures, the level of histone H3 in Over CD was 3-folds that in the KD-CD cells.

**Figure 10:**
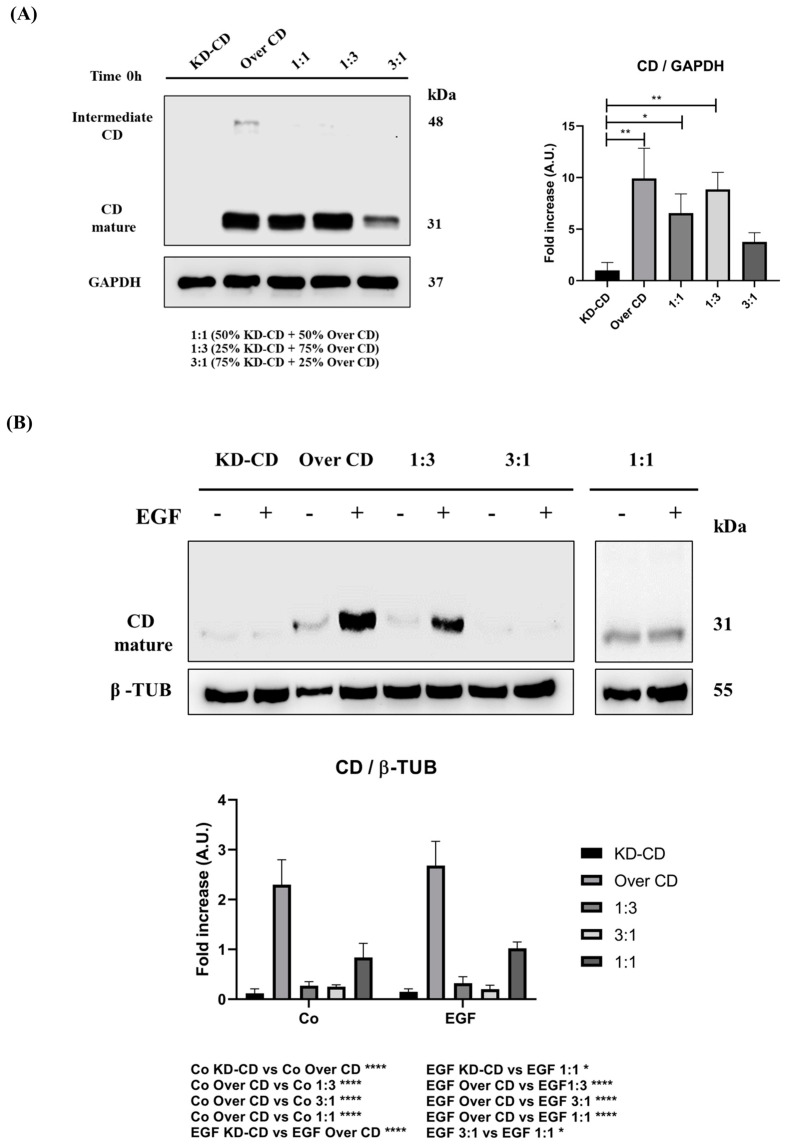
SH-SY5Y Over CD shows a greater ability to grow in suspension compared to KD-CD cells. SH-SY5Y 3D spheroids of pure clones or clones mixed at the indicated ratios were cultured for 7 days in the absence or presence of EGF. Cell homogenates were assayed by Western blotting for CD at time 0 (**A**) and after 7 days (**B**). The filters were stripped and re-probed for GAPDH (**A**) or β-tubulin (**B**) as loading control. Densitometry of the bands is reported in the histograms. One-way ANOVA and two-way ANOVA tests were performed in (**A**) and (**B**), respectively. Significance was considered as follows: **** *p* < 0.0001; ** *p* < 0.01; * *p* < 0.05. “Co” refers to untreated cells (control).

As a confirmation of which subpopulation gained growth advantage in the mixed cultures, we assayed the CD protein content in spheroid’s homogenates (Figure 10). Since in these clones the expression of CD is not subjected to modulation (except for endogenous CD), any change in the content of this protein in the whole culture is largely (if not exclusively) attributable to the proportion of over-expressing versus silenced cells. In the pure KD-CD culture, as well as in the mixed 3:1 culture (where the KD-CD clone is predominant), and to a lesser extent in the mixed 1:1 ratio, an increase in CD content is not observed upon stimulation with EGF, indicating that the absence of CD may reduce the ability of the KD-CD clone to grow in suspension, remaining in a resting phase. By contrast, in the pure Over CD and in the 1:3 mixed culture (containing the Over CD in higher proportion), the increase in CD content is appreciable, indicating that this subclone took advantage for growth in suspension. Densitometric analysis confirms that EGF does not modulate per se the intracellular level of CD (Figure 10B).

### 3.8. Suspended SH-SY5Y Clone Knocked-Down for Cathepsin D Rescues the Ability to Grow in Adherent Condition

**Figure 11:**
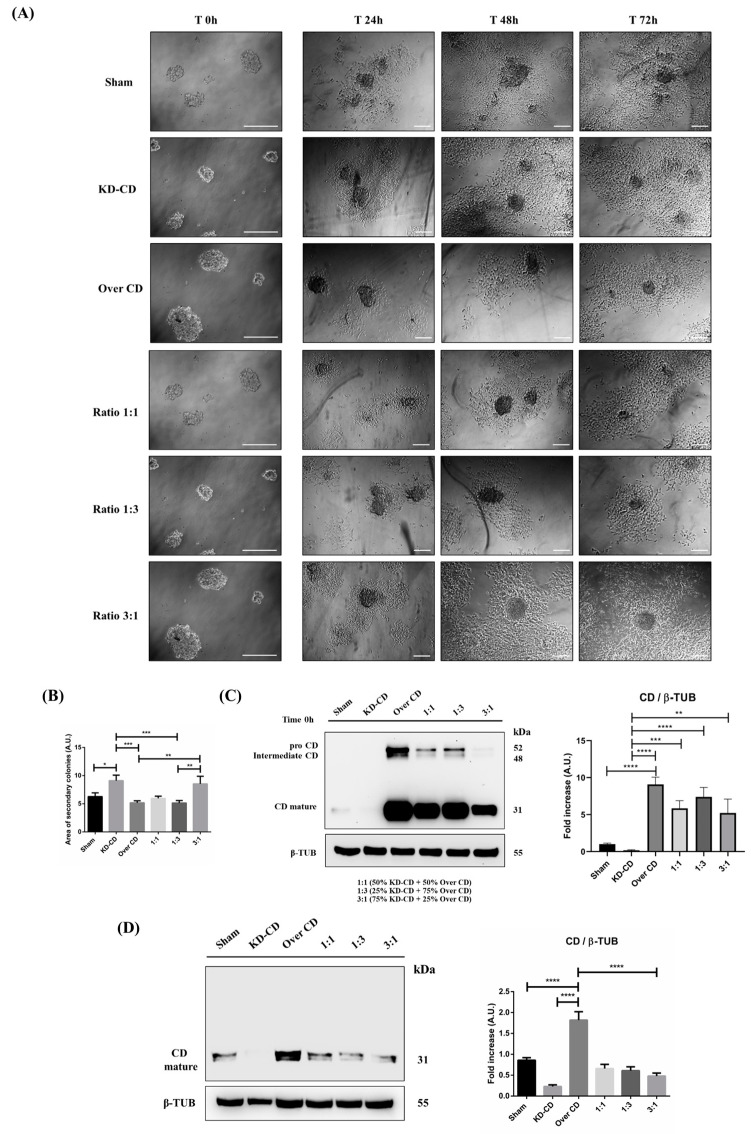
CD knocked-down SH-SY5Y cells rescue the ability to grow in the adherent condition. SH-SY5Y Sham, KD-CD, Over CD and mixed co-cultures, 50% KD-CD + 50% Over CD cells (1:1), 25% KD-CD + 75% Over CD (1:3) and 75% KD-CD + 25% Over CD cells (3:1) were seeded in non-adherent Petri dishes and allowed to grow for 72 h to allow spheroid formation (500,000 cells/Petri). On the third day (indicated in Figure as Time 0 h), neurospheres were collected, resuspended in fresh medium, plated in adherent Petri dishes and maintained in culture for a further 72 h. New fresh medium was replaced every day. Cell homogenates were processed for Western blot analysis. (**A**) Images were acquired at the phase-contrast microscope every day to monitor cell attachment and growth. Scale bar = 100 μm; magnification = 20× (T0 h), 5× (T24 h, 48 h, 72 h). (**B**) Graph representing the area of secondary colonies calculated in different representative fields of three separate experiments (at the endpoint of 72 h). One-way ANOVA test was performed. Significance was considered as follows: *** *p* < 0.001; ** *p* < 0.01; * *p* < 0.05. (**C**,**D**) Western blot analysis of CD in cell homogenates collected at time 0 (**C**) and after 72 h (**D**). The membranes were stripped and re-probed for β-tubulin as loading control. The blots are representative of three independent experiments. Densitometry of the bands is reported in the histograms. One-way ANOVA test was performed. Significance was considered as follows: **** *p* < 0.0001; *** *p* < 0.001; ** *p* < 0.01.

In vivo, small clusters of circulating tumor cells can reach distant organs where they must attach and grow for forming secondary metastasis. To mimic such situations in vitro, the spheroids of suspended cells, from either pure or mixed clones, were placed in culture Petri dishes for attachment and allowed to grow for 72 h. This corresponds to a switch from 3D to the 2D culture condition. The cultures were imaged at 24, 48 and 72 h (Figure 11A).

It is apparent that KD-CD cells, either cultured as pure clone or mixed with Over CD cells, displayed the greatest ability to adhere and grow onto a solid matrix, giving rise to secondary colonies. By contrast, Over CD cells (which were the most actively proliferating in suspension) were less prone to attach and rescue the growth as adherent colonies. The area of the neo-formed secondary colonies was calculated, and it was higher in pure KD-CD and in the mixed 3:1 culture (Figure 11B), confirming that KD-CD cells efficiently rescued the ability to proliferate in the adherent condition. We measured the CD content in the attached colonies as an indirect marker of the prominent subclone in the pure and mixed populations (Figure 11C,D). Whatever the relative proportion in the starting co-culture of spheroids (i.e., at either 1:1, 1:3 or 3:1 ratios), the KD-CD clone became prevalent in the adherent colonies, as indicated by the very low content of CD in the whole homogenates. This confirms that overexpression of CD limits the anchorage-dependent growth of neuroblastoma cells, as also reported in [7].

**Figure 12:**
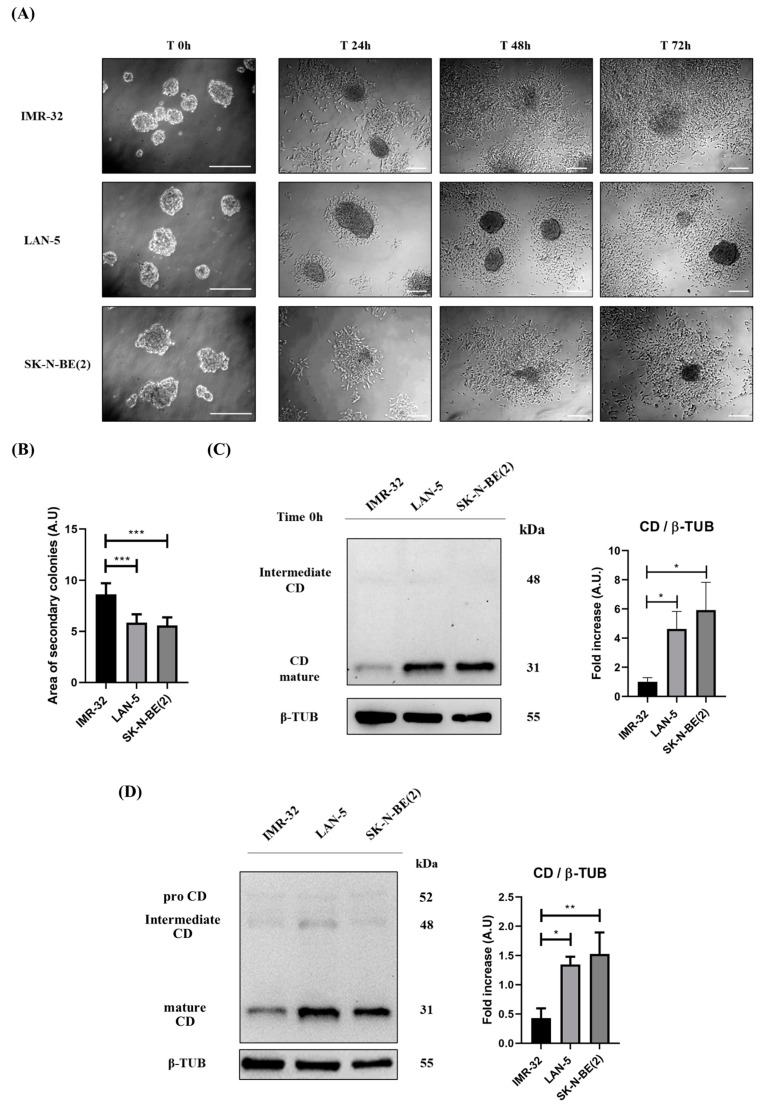
IMR-32 cells expressing low CD levels show a greater ability to grow in the adherent condition. IMR-32, LAN-5 and SK-N-BE(2) cells were seeded in non-adherent Petri dishes and allowed to grow for 72 h to allow spheroid formation (500,000 cells/Petri). On the third day (indicated in Figure as Time 0 h), neurospheres were collected, resuspended in fresh medium, plated in adherent Petri dishes and maintained in culture for a further 72 h. New fresh medium was replaced every day. Cell homogenates were processed for Western blot analysis. (**A**) Images were acquired at the phase-contrast microscope every day to monitor cell attachment and growth. Scale bar = 100 μm; magnification = 20× (T0 h), 5× (T24 h, 48 h, 72 h). (**B**) Graph representing the area of secondary colonies calculated in different representative fields of three separate experiments (at the endpoint of 72 h). One-way ANOVA test was performed. Significance was considered as follows: *** *p* < 0.001. (**C**,**D**) Western blot analysis of CD expression in cell homogenates collected at time 0 (**C**) and after 72 h (**D**). The membranes were re-probed for β-tubulin as loading control. The blots are representative of three independent experiments. Densitometry of the bands is reported in the histograms. One-way ANOVA test was performed. Significance was considered as follows: ** *p* < 0.01; * *p* < 0.05.

Accordingly, in IMR-32 cells, which express low CD levels, the calculated area of neo-formed secondary colonies was higher compared to that of LAN-5 and SK-N-BE(2), characterized by high CD expression (Figure 12A,B). This is further confirmed by looking at the expression of CD in the neurospheres before their transfer to the 2D culture (time zero) and after formation of the colonies at day 3 (Figure 12C,D). Note that IMR-32 cells, which express the lowest CD, form neurospheres that are smaller in size but retain the ability to attach and grow more efficiently on the substrate.

### 3.9. Suspended KD SH-SY5Y Cells Express E-Cadherin When Grown in 2D Adherent Condition

**Figure 13:**
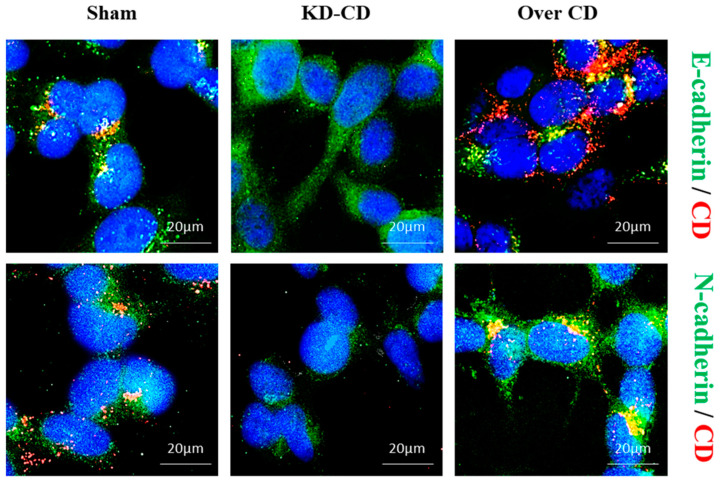
Low expression of Cathepsin D promotes the acquisition of an epithelial phenotype.Sham, KD-CD and Over CD clones were seeded in 3D cultures and allowed to grow for 4 days. Later, 3D spheroids were transferred on to poly-lysine-coated coverslips and allowed to grow for a further 2 days. At the end of the experiment, coverslips were fixed and stained for immunofluorescence double staining of CD (red)–E-cadherin (green) or CD (red)–N-cadherin (green). Nuclei were stained with DAPI. Scale bar = 20 µm; magnification = 63×. Representative images of three independent experiments are shown.

Next, we checked whether the switch from the suspended growth to the substrate-adherent growth was associated with a switch of the membrane adhesion markers epithelial (E) and neural (N) cadherins, a phenotypic change known as the mesenchymal to epithelial transition (MET). When KD-CD and Over CD cells grown as 3D cultures (for 4 days) were allowed to re-adhere (for 2 days) to poly-lysine-coated coverslips, the KD-CD cells rescued the ability to grow in 2D more efficiently than the Over CD cells. This was associated with the (epigenetic) change in the expression of the cell adhesion cadherin type. In fact, KD-CD cells promptly acquired a substrate-attached phenotype, as indicated by high E-cadherin together with low N-cadherin expression, while Over CD cells still retained a typical “suspended” phenotype characterized by strong N-cadherin expression and low E-cadherin expression (Figure 13).

### 3.10. CTSD Expression Is Negatively Correlated with miRNAs Involved in Cell Adhesion and Migration

**Figure 14:**
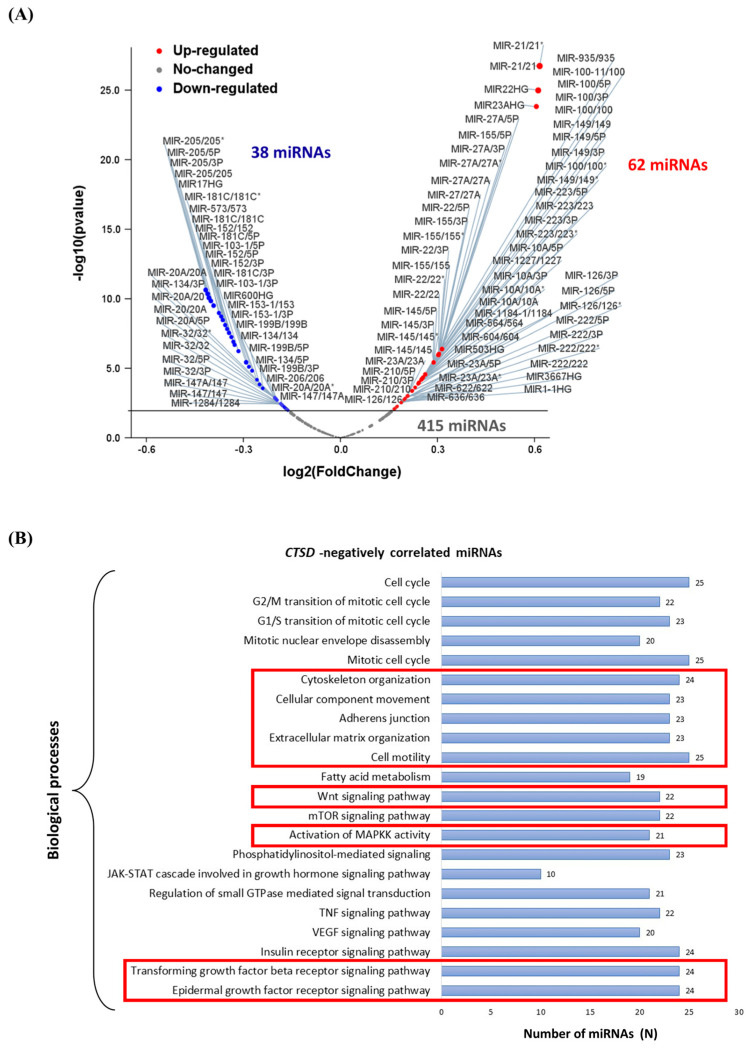
*CTSD* negatively correlated microRNA-modulated biological processes and pathways. (**A**) Volcano plot displaying the differentially expressed miRNAs. Red dots represent *CTSD* positively correlated miRNAs, while blue dots represent *CTSD* negatively correlated miRNAs. (**B**) Graph reporting the *CTSD* negatively correlated miRNAs biological processes.

The above findings indicate that in NB cells, CD plays a dual role in adherent 2D culture versus non-adherent 3D culture. We hypothesized that *CTSD* gene could be regulated via epigenetic mechanisms involving non-coding RNAs. This could have an impact on metastasization and the patient’s clinical outcome. To address this issue, we performed the bioinformatic analysis in NB patients (data extracted from TCGA) to study the miRNAs correlated with *CTSD* expression. We identified the differentially expressed miRNAs based on a correlation coefficient value >0.25 (for upregulated, i.e., positively correlated with *CTSD* expression) or <−0.25 (for downregulated, i.e., negatively correlated with *CTSD* expression) with adjusted *p*-value < 0.001. Out of a total 515 miRNAs, 62 miRNAs were upregulated and 38 were downregulated, as shown in the Volcano plot (Figure 14A). Interestingly, the 38 miRNAs negatively correlated with *CTSD* expression are involved in the regulation of cytoskeleton organization, cell adhesion, cellular component movement, cell motility, activation of MAPK activity, epidermal growth factor receptor, transforming growth factor beta receptor signaling and Wnt signaling pathways (Figure 14B).

Next, we performed an in silico transcriptomic analysis of the genes correlated to *CTSD* expression. We retrieved the RNA-seq data (mRNA expression profile from the TCGA database TARGET, 2018) and performed a co-expression analysis to identify the most significant differentially expressed genes (DEGs) that were positively (upregulated genes) and negatively (downregulated genes) correlated with *CTSD* expression in neuroblastoma patients’ samples. Notably, the main biological processes regulated by the genes negatively correlated with *CTSD* included calcium-dependent cell–cell adhesion or homophilic cell adhesion, regulation of mesenchymal cell proliferation, cadherin signaling pathway and Wnt signaling pathway (Supplementary Figure S4A).

To further understand the clinical relevance of the 38 miRNAs targeting CD in NB pathogenesis and progression, we searched the TCGA database for the presence of altered expression in human samples. The oncoprint relative to 20 miRNAs of interest in 248 patients is shown in Supplementary Figure S4B. The most relevant miRNAs involved in regulation of cytoskeleton organization, cell adhesion, cellular component movement, cell motility and Wnt signaling pathway are reported in oncoprint (namely, MIR-124-1/3P, MIR-124-1/5P, MIR-147/147A, MIR17HG, MIR-181C/3P, MIR-181C/5P, MIR-194-2/5P, MIR-195/5P, MIR-199B/3P, MIR-199B/5P, MIR-205/5P, MIR-206/206, MIR-20A/5P, MIR-32/3P, MIR-32/5P, MIR-573/573, MIR-99A/3P, MIR-99A/5P, MIR-32/3P and MIR-32/5P). *CTSD* and miRNAs were not affected by any genetic alterations. It is evident that with a few exceptions, most of the NB patients analyzed express low or very low levels of *CTSD* mRNA. Supplementary Figure S4B also reports the heatmap depicting the *CTSD* mRNA expression correlated with miRNAs expression.

## 4. Discussion

Neuroblastoma is the most common extracranial solid tumor of childhood, responsible for over 15% of cancer-related deaths [2,16]. Despite recent advances in a multimodal therapeutic strategy, including EGFR targeting, NB continues to cause high mortality. NB tumorigenesis and metastasization are driven by the abnormal activity of oncogenic signaling pathways driving cell proliferation, cell survival and cell motility. A better understanding of the regulatory mechanisms of downstream growth factor receptors may help the development of novel targets and effective therapeutics. Based on bioinformatic analyses of public datasets, we have previously demonstrated that high expression of the lysosomal protease CD is a predictor of good prognosis in NB patients bearing high levels of *EGFR* [7], and here, we show that NB patients with high expression of *CTSD* present with a better clinical outcome when combined with low rather than with high *MYCN* expression. Interestingly, *MYCN* and *EGFR* were negatively correlated, while *CTSD* and *MAPK1* (which codes for ERK1, down-stream to EGFR signal) were positively correlated. The in silico data were confirmed and further corroborated mechanistically with in vitro studies. EGF reduced CD expression in association with the induction of cell proliferation in *MYCN*-not-amplified SH-SY5Y cells, while *MYCN*-amplified neuroblastoma cell lines IMR-32, LAN-5, SK-N-BE(2) were insensitive to EGF. Noteworthy, transgenic Over CD SH-SY5Y cells are less sensitive to EGF and present lower level of the down-stream ERK1 transducer compared to KD-CD cells. This could result from either proteolytic events (either direct or indirect) within the endosome or from epigenetic events associated with clone selection. CD is the unique aspartic protease ubiquitously expressed in all cell types, and it has been demonstrated to play a fundamental role in physiology and pathology in human and non-human species [17,18,19,20]. We speculate that forcing the KD or over-expression of CD alters the whole metabolome of the cell because of its proteolytic role within the endosomal–lysosomal compartments (with an impact on autophagy-mediated homeostasis) and in the extracellular environment. This hypothesis, which is supported by the data here obtained with PstA, is presently under testing in our laboratory.

The conventional 2D cellular model does not adequately resemble the complexity of the tumor mass. Cells grown as 3D aggregates (multicellular spheroids) much more closely recapitulate the in vivo structure of cancers and possess features in common with primary tumors, including cells in different proliferative and metabolic states, also due to growth factor, nutrient and oxygen availability [8,12,21,22,23,24,25,26]. Important phenotypic and metabolic differences, including migration, proliferation and response to toxic drugs, have been reported when comparing the NB cells cultured in adhesion as a 2D monolayer or in suspension as 3D spheroids [25,26,27,28,29].

Furthermore, 3D multicellular spheroids resemble the circulating clusters of metastatic cancer clones. To metastasize, tumor cells detach from the basement membrane, but not all floating cells survive in the fluid environment (blood and lymph). We sought to understand whether and how CD differentially impacts the growth of attached (2D) or suspended (3D) NB cells under EGF stimulation. We took advantage of transgenic neuroblastoma SH-SY5Y cells either knocked-down for (KD-CD) or overexpressing CD (Over CD) available in our laboratory to address this issue. This cell line is *MYCN*-not-amplified and responsive to EGF. The type of culture system influences the proteome [30]. Changes in signaling cascades, like PI3K/AKT/mTOR and EGFR/MAPK, two central regulators of cell growth, survival and metabolism, have been well documented [22,23,31]. Strikingly, we found that overexpression of CD while inhibiting NB growth in 2D cultures was instead beneficial for the growth in 3D. Accordingly, wild-type SH-SY5Y cells enhanced the expression of CD when the culture was switched from the adherent to suspended condition. As a preliminary observation, we found that in Over CD cells grown in 3D, the ERK1/2 signaling was upregulated, and it was not further stimulated by EGF (Supplementary Figure S5). These data need, however, to be corroborated with more experiments. Consistently, EGF only slightly stimulated the proliferation rate of the transgenic Over CD clone grown in 3D cultures. This further supports the view that CD is one of the main drivers of cell proliferation control in neuroblastomas.

The implications of neuroblastoma intratumor heterogeneity in cancer cell proliferation and metastasization and clinical outcome remain elusive [32,33].

Within the tumor context, normal and cancer cells with different genetic and epigenetic backgrounds dynamically compete for space and survival, metabolic substrates, and the fittest clones will eventually expand by eliminating the less fitting ones [34]. To mimic the tumor heterogeneity arising from clonal evolution that could lead to clones expressing CD at different levels, we tested the growth ability of mixtures at different ratios of the two clones. Briefly, it was found that the KD-CD clone surpassed the Over CD clone when the cells were co-cultured in 2D, and vice versa, the Over CD clone surpassed the KD-CD clone when the cells were co-cultured in a suspension. We exploited our CD-engineered clones to understand the role of CD in culture conditions that recapitulate in vitro the steps of metastatic spreading, that is, the transition from adherent to suspended to adherent growth. Briefly, we found that upregulation of CD expression is necessary to guarantee the survival and proliferation of the cells in suspension, while it is necessary to downregulate its expression to allow adherence and anchorage-dependent growth of the tumor cells. Consistently, re-expression of E-cadherin was observed in KD-CD neurospheres placed on a substrate, while the Over CD neurospheres were still expressing N-cadherin 48 h after plating on a substrate. These data indicate that KD-CD are more prone to revert to their mesenchymal-to-epithelial like phenotype.

We can hypothesize that CD expression is epigenetically modulated during the metastatic cascade of neuroblastoma cells. The bioinformatic analyses here reported support this hypothesis. The *CTSD* gene can be regulated via epigenetic mechanisms involving non-coding RNAs. For example, miR-185-3p directly targets *CTSD* in gastric cancer [35], and miR-3619 targets both *CTSB* and *CTSD* in their 3′ UTR sequence to negatively regulate their expression [36]. High levels of miR-3619-3p are associated with enhanced migratory and invasive ability of papillary thyroid carcinoma cells. MiR-3619-3p was found highly expressed in metastatic papillary thyroid cancer tissues [37] and upregulated in recurrent esophageal carcinoma tissue samples, compared to non-recurrent esophageal carcinoma [38]. *CTSD* could also be indirectly regulated by non-coding RNAs. *CTSD* gene transcription is enhanced by ERα, one of the estradiol (E2)-activated transcription factors. The coding gene *ESR1* is a direct target of miR-301a-3p, and this interaction indirectly leads to the downregulation of *CTSD* expression in breast cancer [39]. In addition, miR-204 downregulates *M6PR*, *IGF2R* genes involved in the lysosomal segregation of cathepsin B and cathepsin D lysosomal enzymes in medulloblastoma cells [40]. Here, we found at least 38 miRNAs able to downregulate CD in human neuroblastomas, and these miRNAs were associated with biological processes involved in cell migration and cell adhesion, among others. Of course, we cannot exclude other mechanisms of CD regulation at transcriptional or translational levels.

## 5. Conclusions

**Figure 15:**
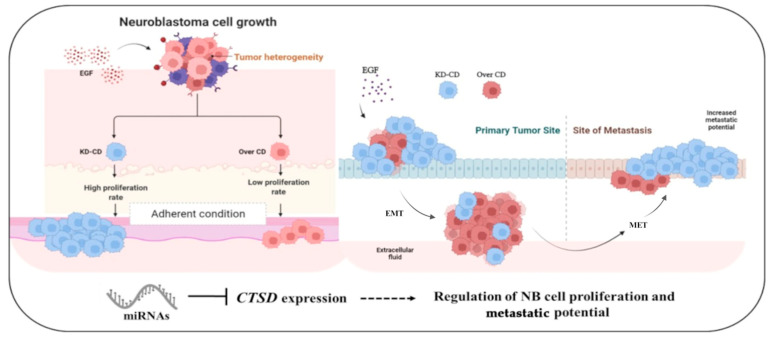
The cartoon summarizes in graphical form the findings here reported.

The cartoon in Figure 15 summarizes our findings. Here, we demonstrate that CD plays a dual role in the control of cell proliferation depending on whether the cells are growing on substrate or in suspension. To our knowledge, this is the first evidence for such a role of CD in neuroblastomas. Another important novelty of the present work is the use of mixed clones expressing CD at different levels, a bona fide mimic of intratumor neuroblastoma heterogeneity. In our model, CD-silenced clones showed better fitting for adherent growth, while CD-overexpressing clones were better fitting for suspended (non-adherent) growth. Interestingly, when the mixed neurospheres were put back to grow as adherent cells again, the CD-silenced cells gained advantage for growth. This is the first experimental model for mimicking in vitro tumor heterogeneity possibly resulting from genetic or epigenetic alterations during tumorigenesis and clonal evolution. Of course, this model should be taken as a proof-of-concept. It is likely that tumors can epigenetically regulate CD depending on whether the cells must survive in the mesenchymal space and body fluids, as in the early step of metastasis, or must grow adherent to the matrix, as in the late step of colony formation.

Collectively, we have uncovered a novel function of CD in the metastatic spreading of tumors. This finding may have translational relevance, and we propose CD as a biomarker for metastatic neuroblastomas and for the stratification of patients in view of personalized medicine. Further to be considered, the epigenetic modulation of CD expression could be a valuable complementary strategy for preventing NB metastasis.

## Data Availability

Data are contained within the article and supplementary materials.

## Supplementary Materials

The following supporting information can be downloaded at: https://www.mdpi.com/article/10.3390/cancers16071343/s1, Figure S1: CD knockdown clone expressing high levels of Ki-67 and reduced levels of p27 overtakes Over CD clone in EGF-stimulated growth of mixed cultures; Figure S2: Monitoring of spheroid’s formation in MYCN-amplified neuroblastoma cell lines IMR-32, LAN-5 and SK-N-BE(2) in the presence of EGF and/or PstA; Figure S3: Analysis of histone H3 expression in SH-SY5Y 3D spheroids of pure and mixed clones cocultured in the absence or presence of EGF; Figure S4: CTSD gene expression negatively correlated genes-modulated biological processes and pathways; Figure S5: Analysis of p-ERK1/2 phosphorylation in SH-SY5Y 3D spheroids of KD-CD and Over CD clones challenged with EGF. The original western blots are provided in a separate PDF file.
