# Supplementary material for: Differential Competitive Growth of Transgenic Subclones of Neuroblastoma Cells Expressing Different Levels of Cathepsin D Co-Cultured in 2D and 3D in Response to EGF: Implications in Tumor Heterogeneity and Metastasis
Source: Cancers (Basel). 2024 Mar 29;16(7):1343. doi: 10.3390/cancers16071343 (PMC11010890; doi:10.3390/cancers16071343)
Supplement: Supplementary file 1 [file cancers-16-01343-s001.zip › cancers-2864349-supplementary figures.pdf]

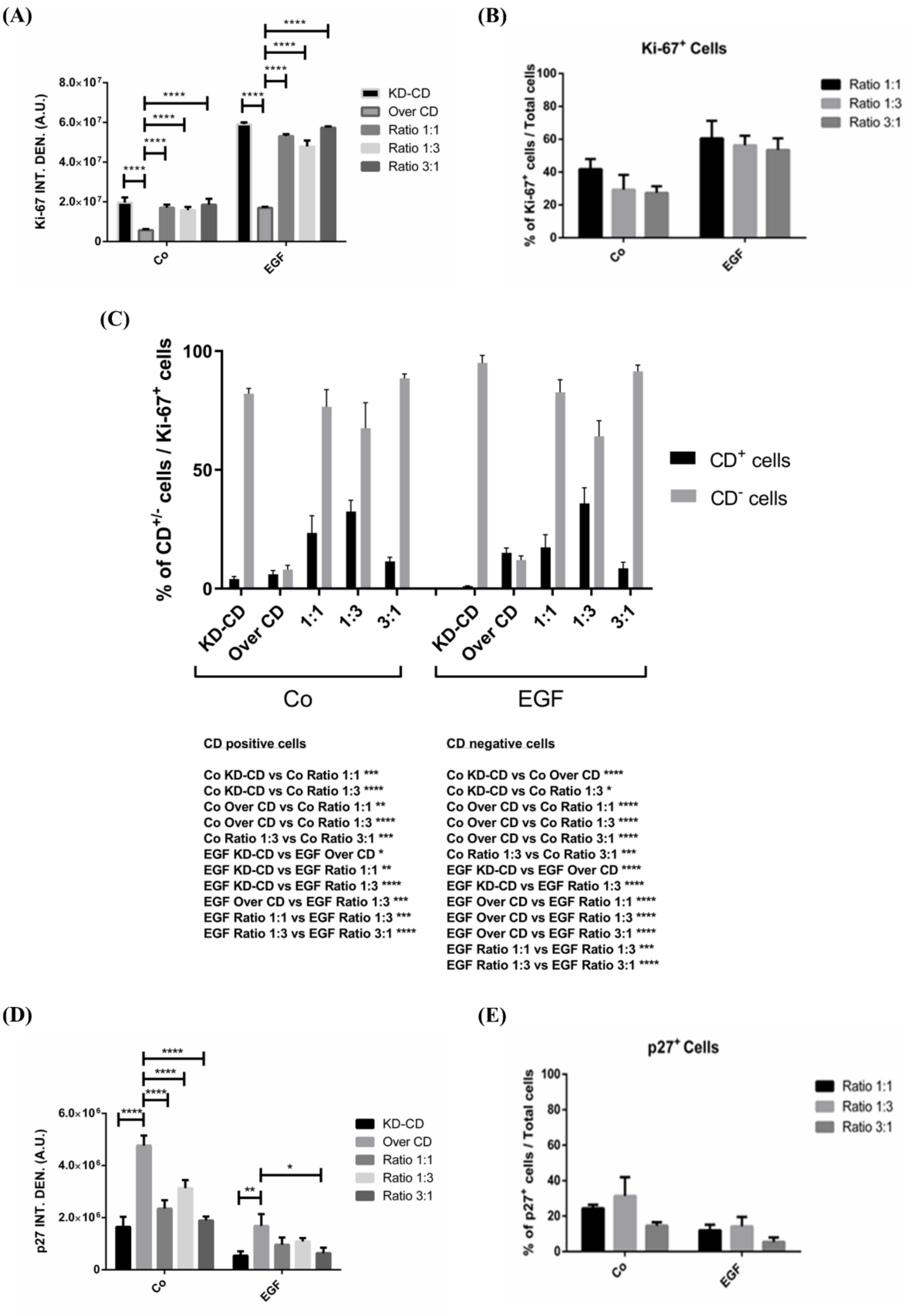

Supplementary Figure S1. **CD knockdown clone expressing high levels of Ki-67 and reduced levels of p27 overtakes Over CD clone in EGF-stimulated growth of mixed cultures.** SH-SY5Y KD-CD, Over CD and mixed cultures at different ratios (50% KD-CD + 50% Over CD cells (1:1), 25% KD-CD + 75% Over CD (1:3)

and 75% KD-CD + 25% Over CD cells (3:1)) were cultured for 72 hours in the presence or not of EGF. At the end of the treatment, cells were fixed and stained for Ki-67-CD and p27-CD. A) Quantification of Ki-67 fluorescent signal was performed by ImageJ software. B) Graph reporting the percentages of Ki-67$^+$ cells relative to the total cell population. C) Graph reporting the percentages of CD$^-$ and CD$^+$ cells relative to the total number of Ki-67$^+$ cells. D) Quantification of p27 fluorescent signal was performed by ImageJ software. E) Graph reporting the percentages of p27$^+$ cells relative to the total cell population. Two-way ANOVA test was performed in all the statistical analysis reported in the graphs. Significance was considered as follow: **** $p < 0.0001$; *** $p < 0.001$; ** $p < 0.01$; * $p < 0.05$.

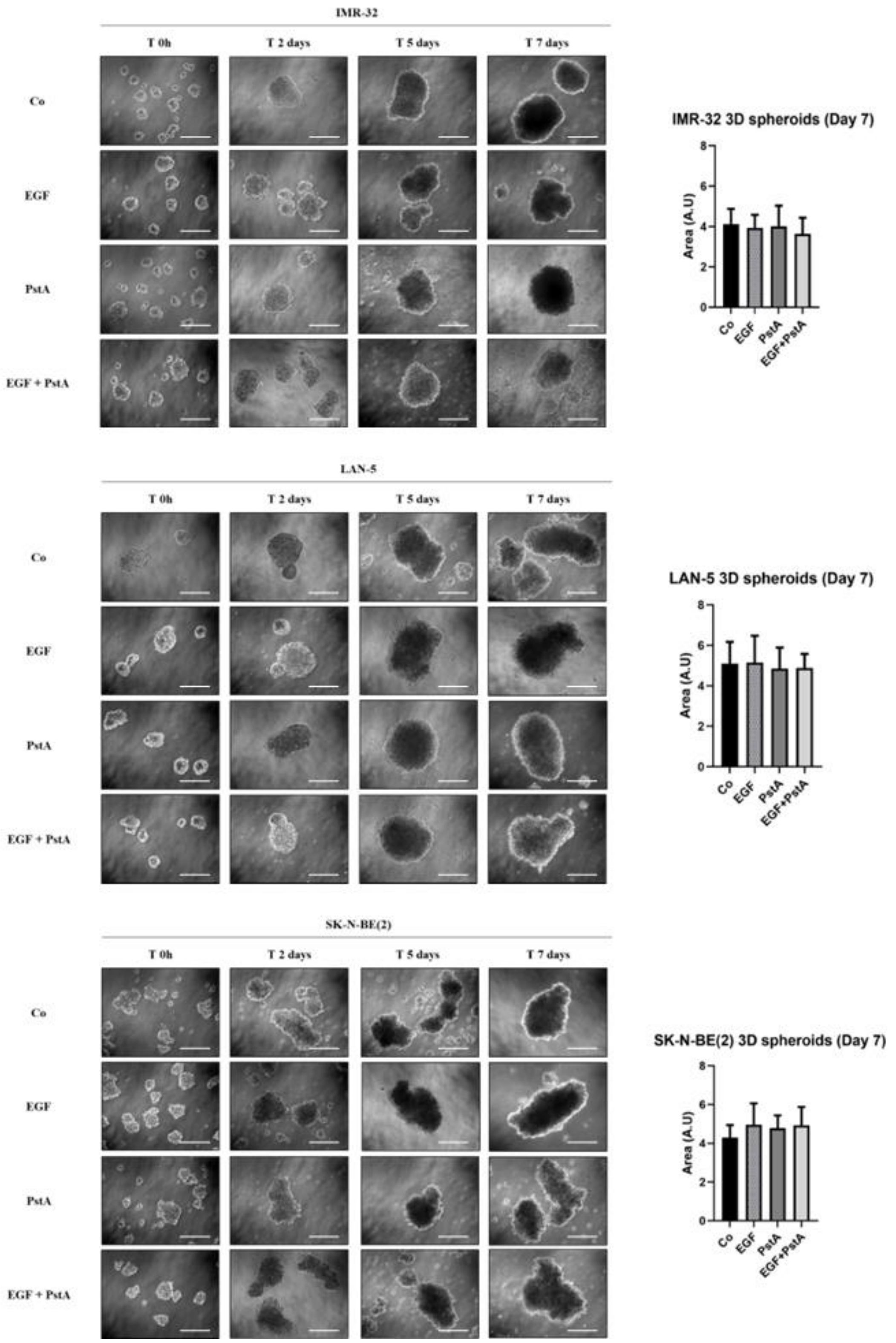

Supplementary Figure S2. **Monitoring of spheroid's formation in *MYCN*-amplified neuroblastoma cell lines IMR-32, LAN-5 and SK-N-BE(2) in the presence of EGF and/or PstA.** IMR-32, LAN-5 and SK-N-BE(2) cells were plated on non-adherent Petri dishes and let grow for 48 hours to allow spheroid's formation. Cells were cultured for 7 days after the first treatment. At time 0h, cells were incubated with EGF and/or PstA and re-treated in fresh medium at day 2 and 5 until the endpoint of 7 days. In the co-treatment condition, PstA was

added before EGF administration to inhibit the enzymatic activity of CD. The 3D spheroid's growth was monitored at the phase-contrast microscope and images were acquired at different time points (time 0h, 2-, 5- and 7-days). Scale bar=100 μm; magnification = 20x. Quantification of spheroid's size was obtained through ImageJ software. The area is indicated as Arbitrary Unit (A.U.). Data represent the average ± S.D. calculated for at least 5 to 10 spheroids for each condition in three separate experiments.

(A)

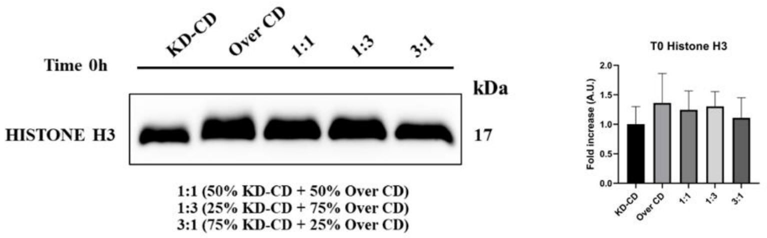

(B)

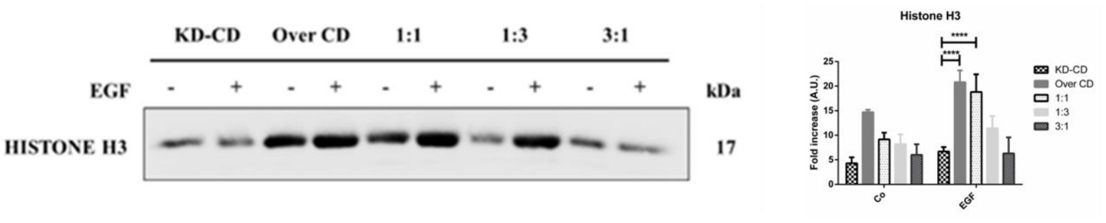

Supplementary Figure S3. **Analysis of histone H3 expression in SH-SY5Y 3D spheroids of pure and mixed clones cocultured in the absence or presence of EGF.** SH-SY5Y 3D spheroids of pure clones or clones mixed at the indicated ratio were cultured for 7 days in the absence or presence of EGF, samples were collected at time 0 (A) and after 7 days (B) and cell homogenates were assayed by western blotting for H3 protein. Densitometry of the bands is reported in the histograms. Significance was considered as follow: **** $p < 0.0001$.

(A)

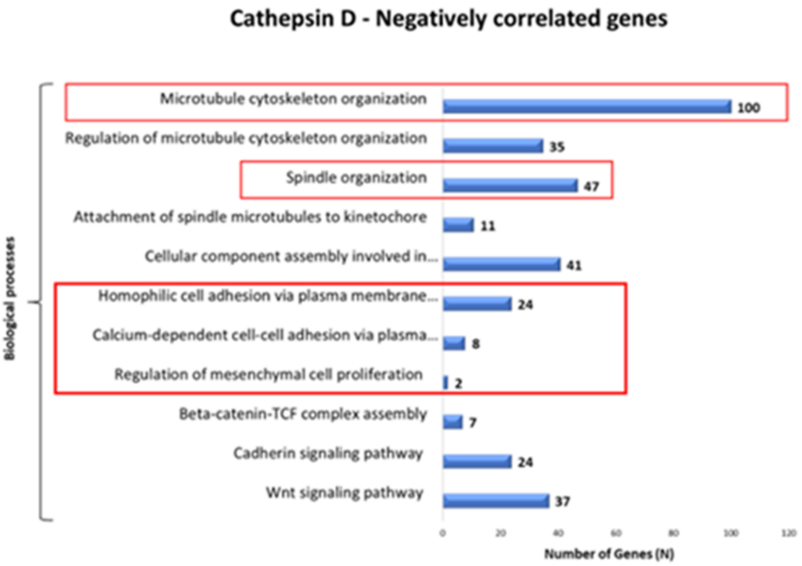

(B)

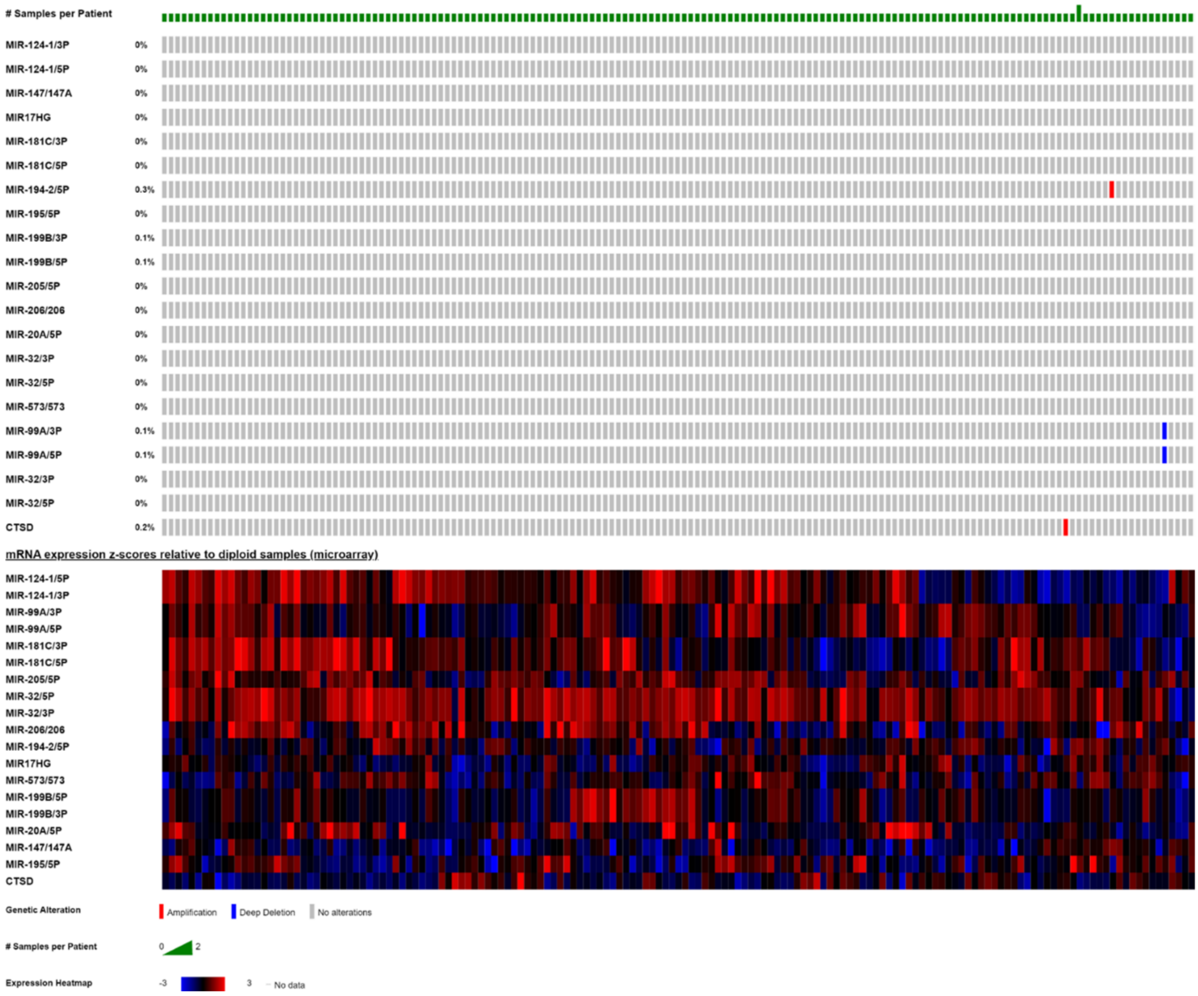

***Supplementary Figure S4. CTSD* gene expression negatively correlated genes-modulated biological processes and pathways.** A) Graph reporting the *CTSD* - negatively correlated genes biological processes. B) Oncoprint relative to 20 miRNAs of interest (involved in regulation of cytoskeleton organization, cell

adhesion, cellular component movement, cell motility, and Wnt signaling pathway) in 248 NB patients. Heatmap displaying the *CTSD* mRNA expression correlated with miRNAs expression.

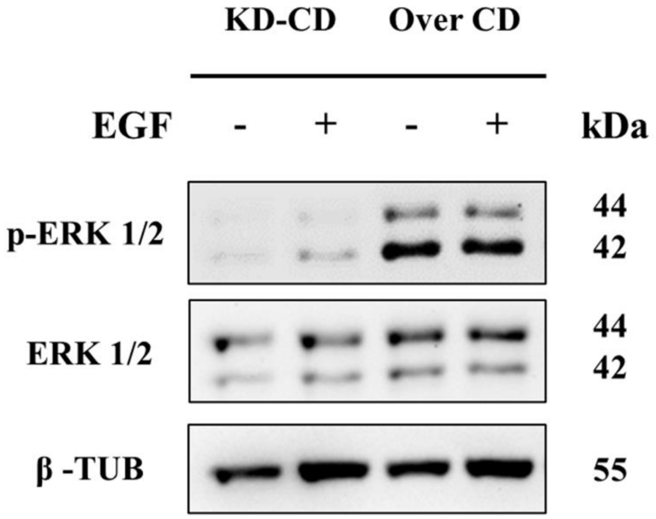

Supplementary Figure S5. **Analysis of p-ERK1/2 phosphorylation in SH-SY5Y 3D spheroids of KD-CD and Over CD clones challenged with EGF.** Western Blotting showing the phosphorylation of ERK 1/2 (p-ERK 1/2) in SH-SY5Y 3D spheroids of KD-CD and Over CD cells cultured in the absence or presence of EGF for 7 days. Samples were processed for western blot analysis of p-ERK 1/2 and total ERK 1/2. Membranes were probed with β-tubulin as loading control.
