# Supplementary material for: Differential Competitive Growth of Transgenic Subclones of Neuroblastoma Cells Expressing Different Levels of Cathepsin D Co-Cultured in 2D and 3D in Response to EGF: Implications in Tumor Heterogeneity and Metastasis
Source: Cancers (Basel). 2024 Mar 29;16(7):1343. doi: 10.3390/cancers16071343 (PMC11010890; doi:10.3390/cancers16071343)

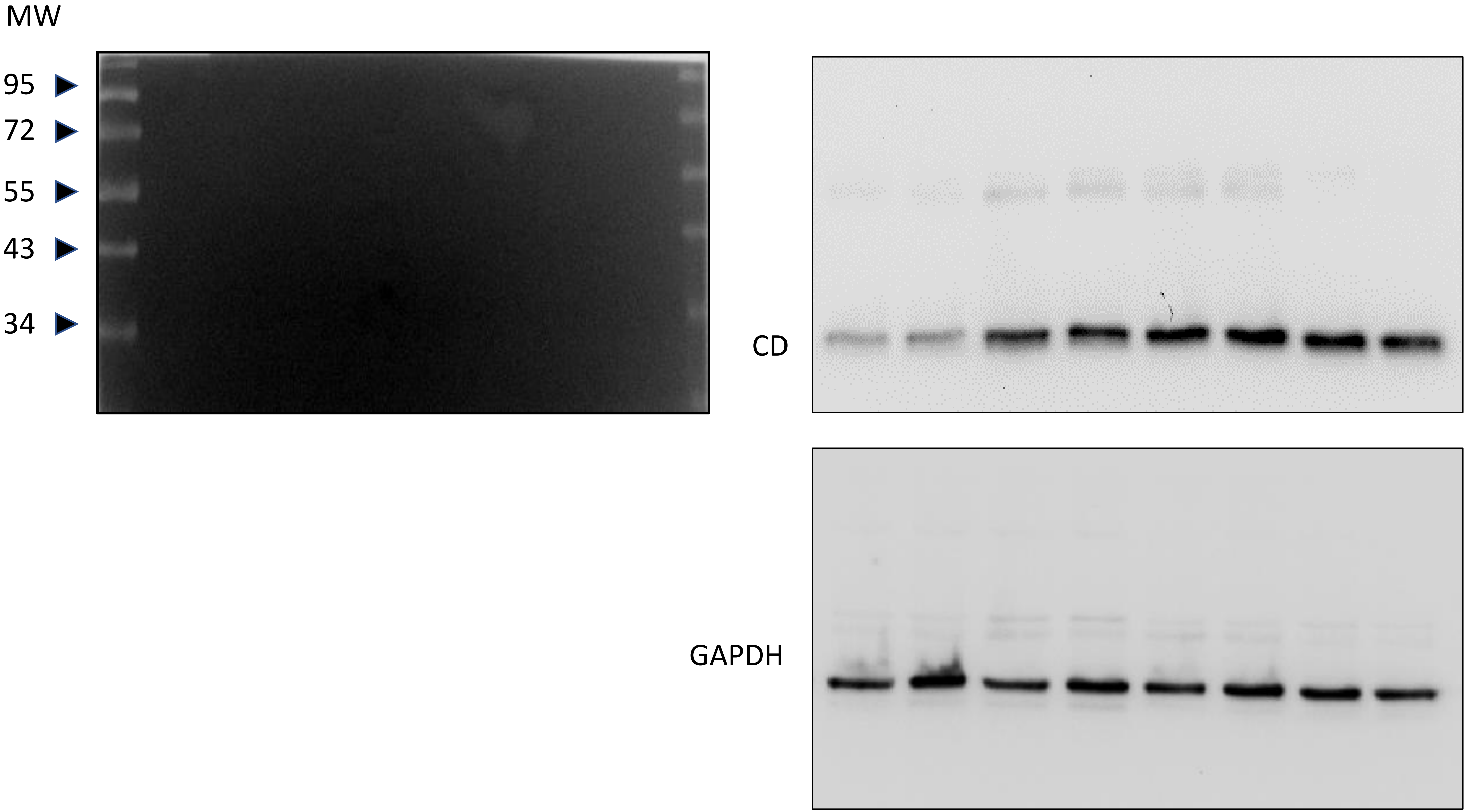

Figure 3A

Figure 4A

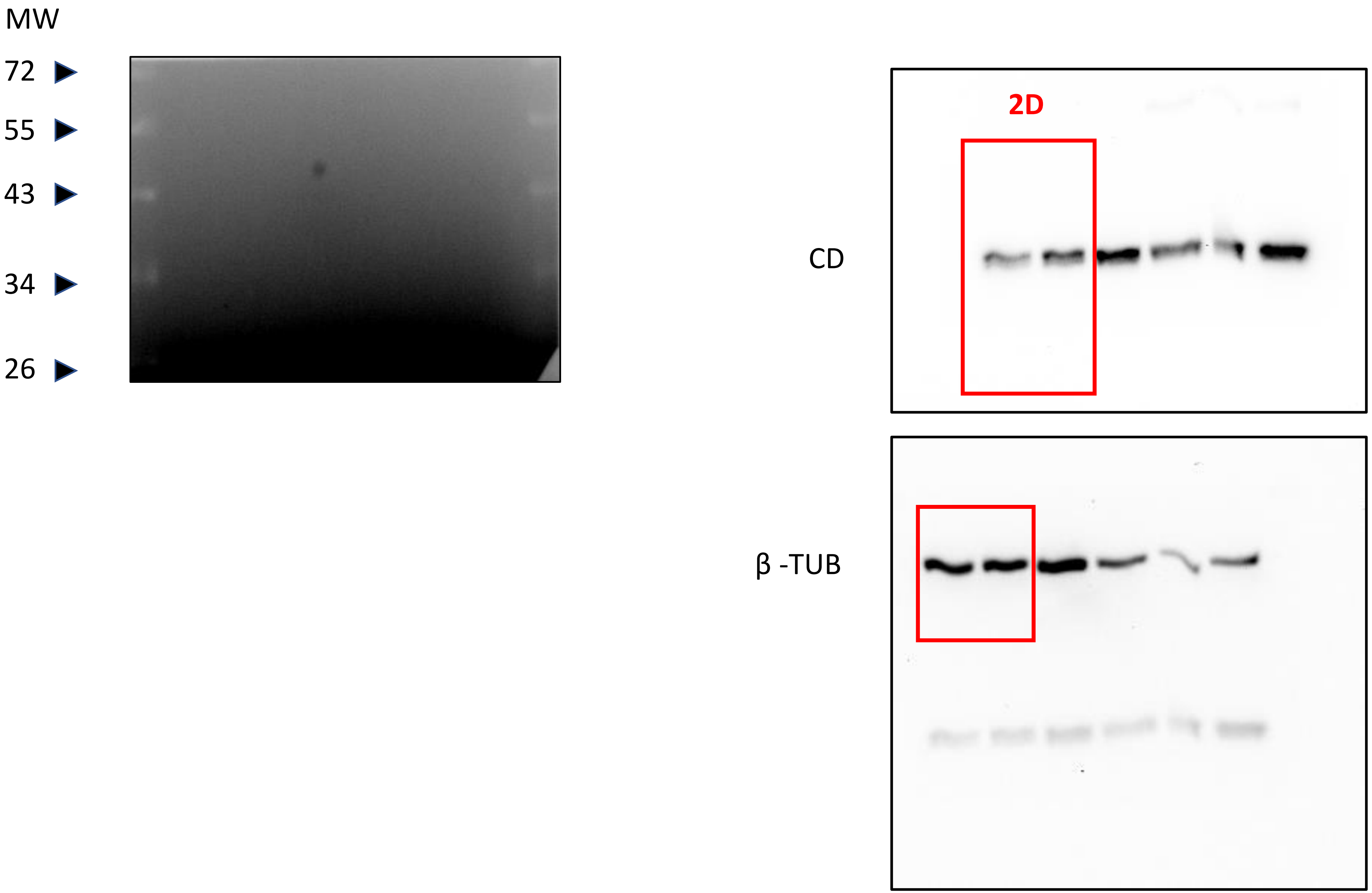

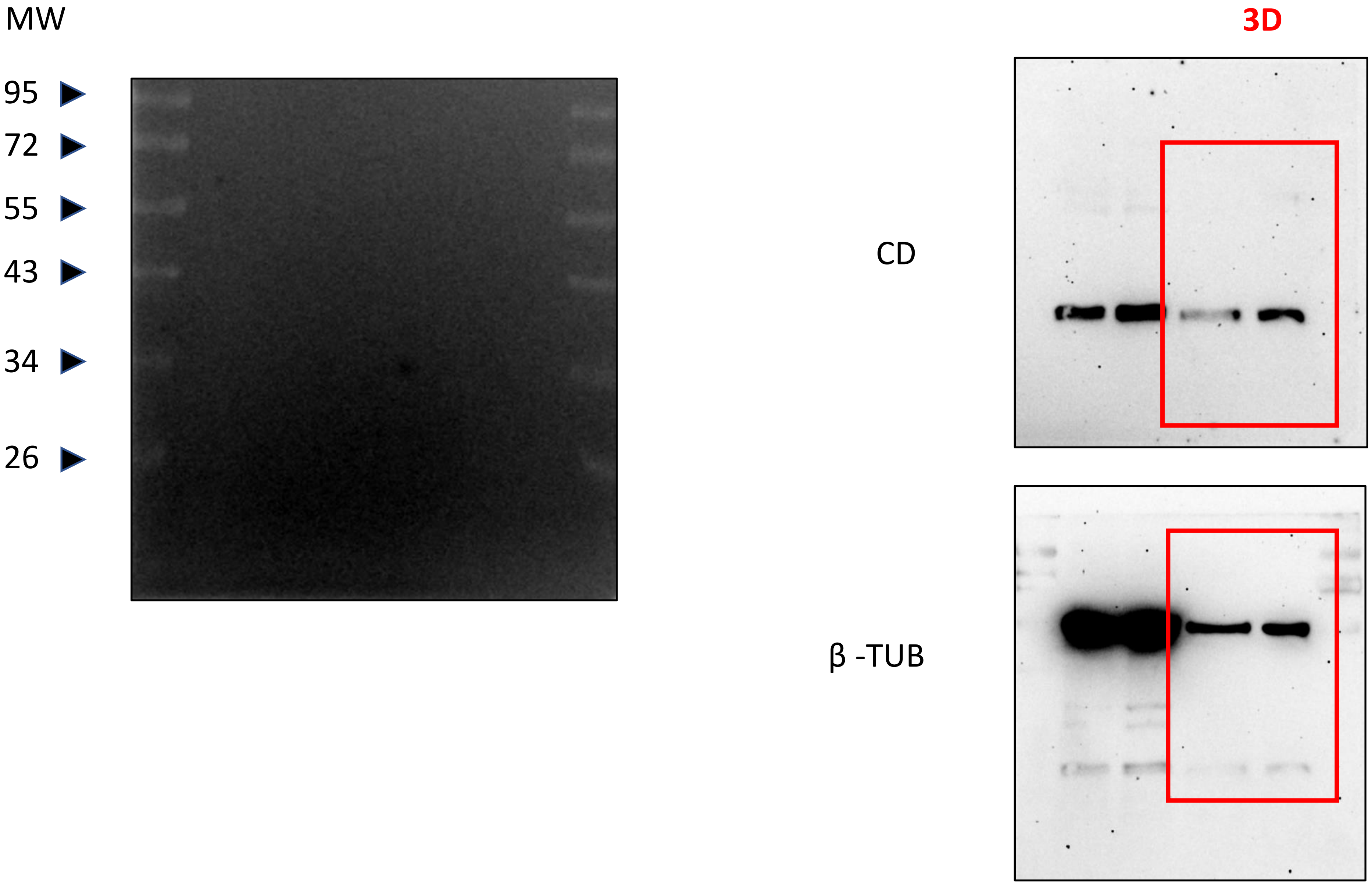

Figure 4A

Figure 5B

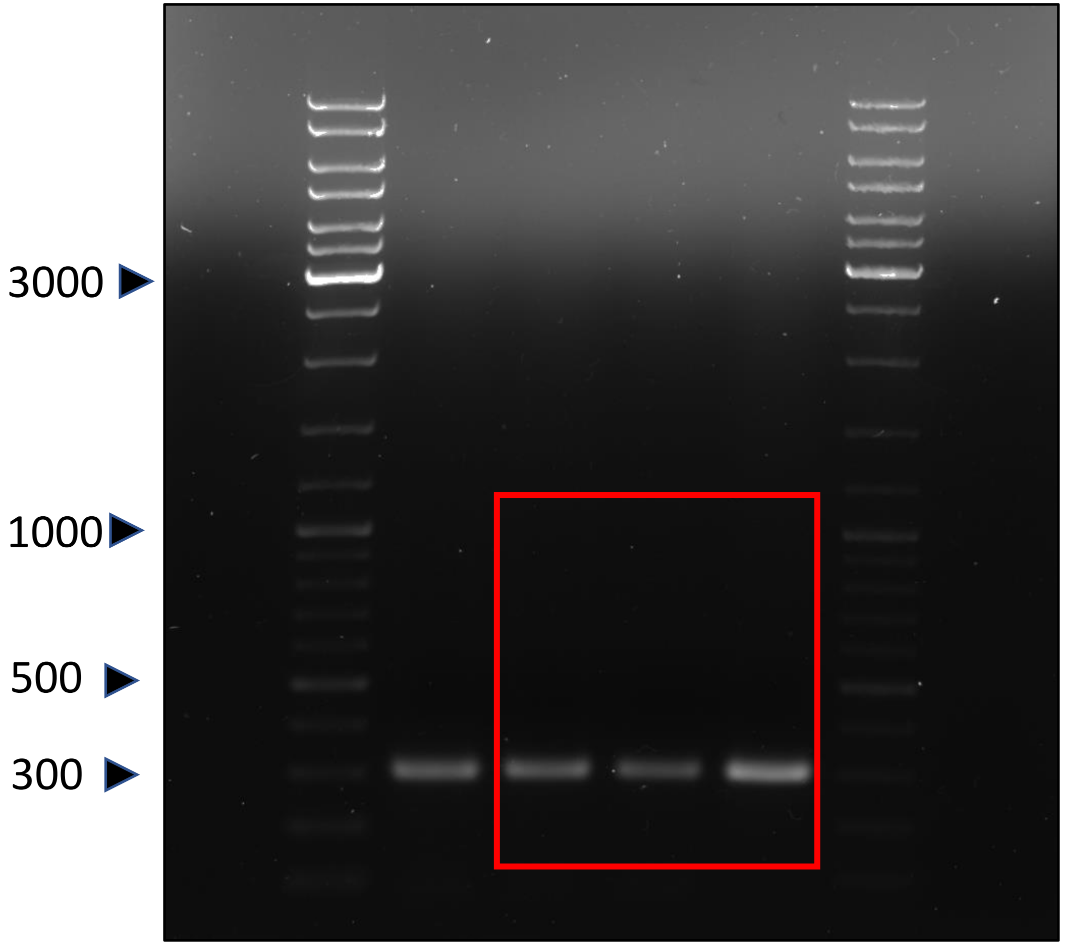

CD

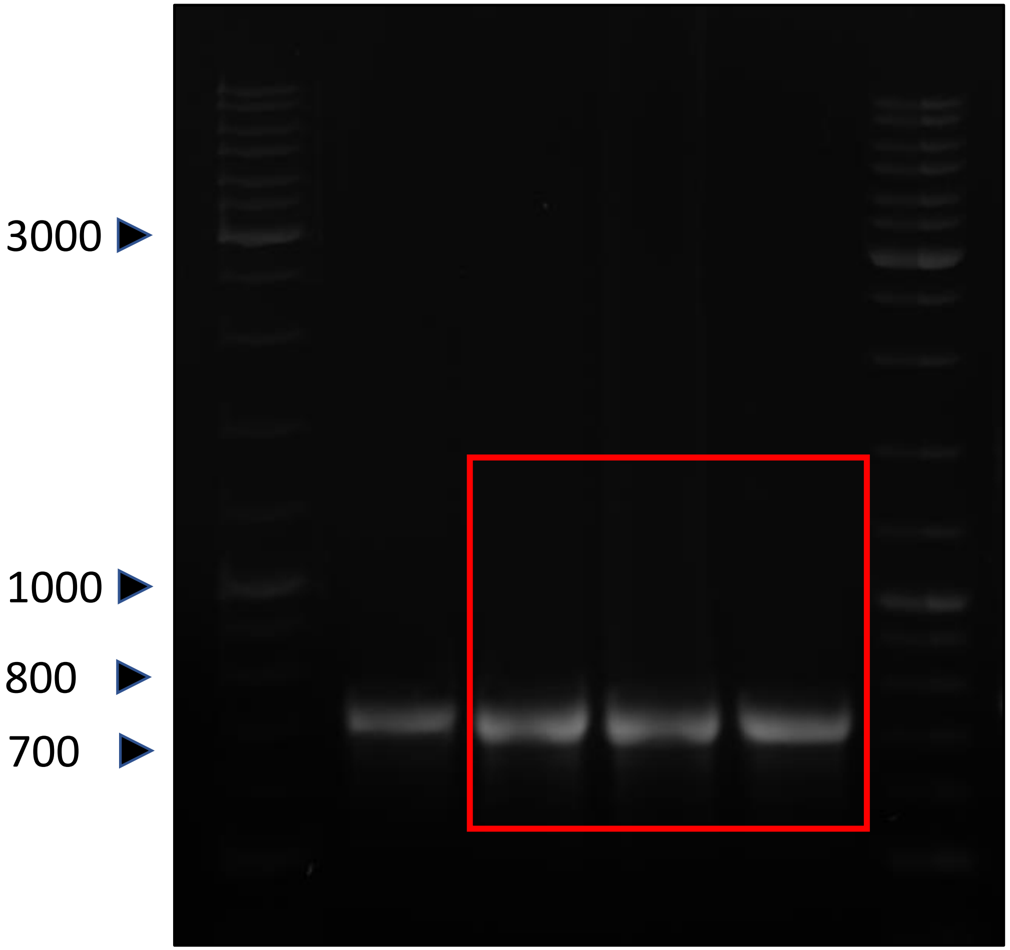

β -ACTIN

Figure 5C

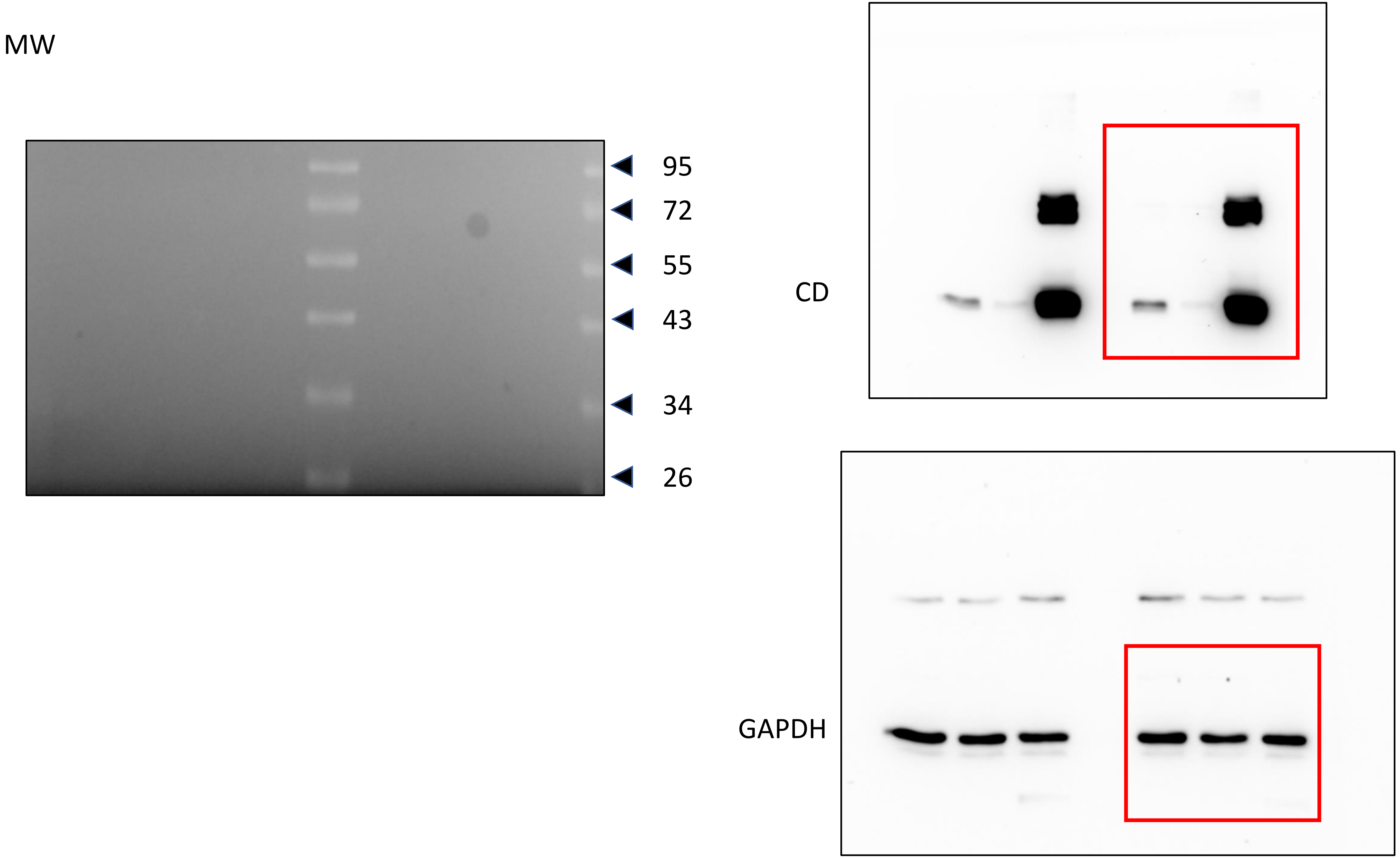

Figure 6C

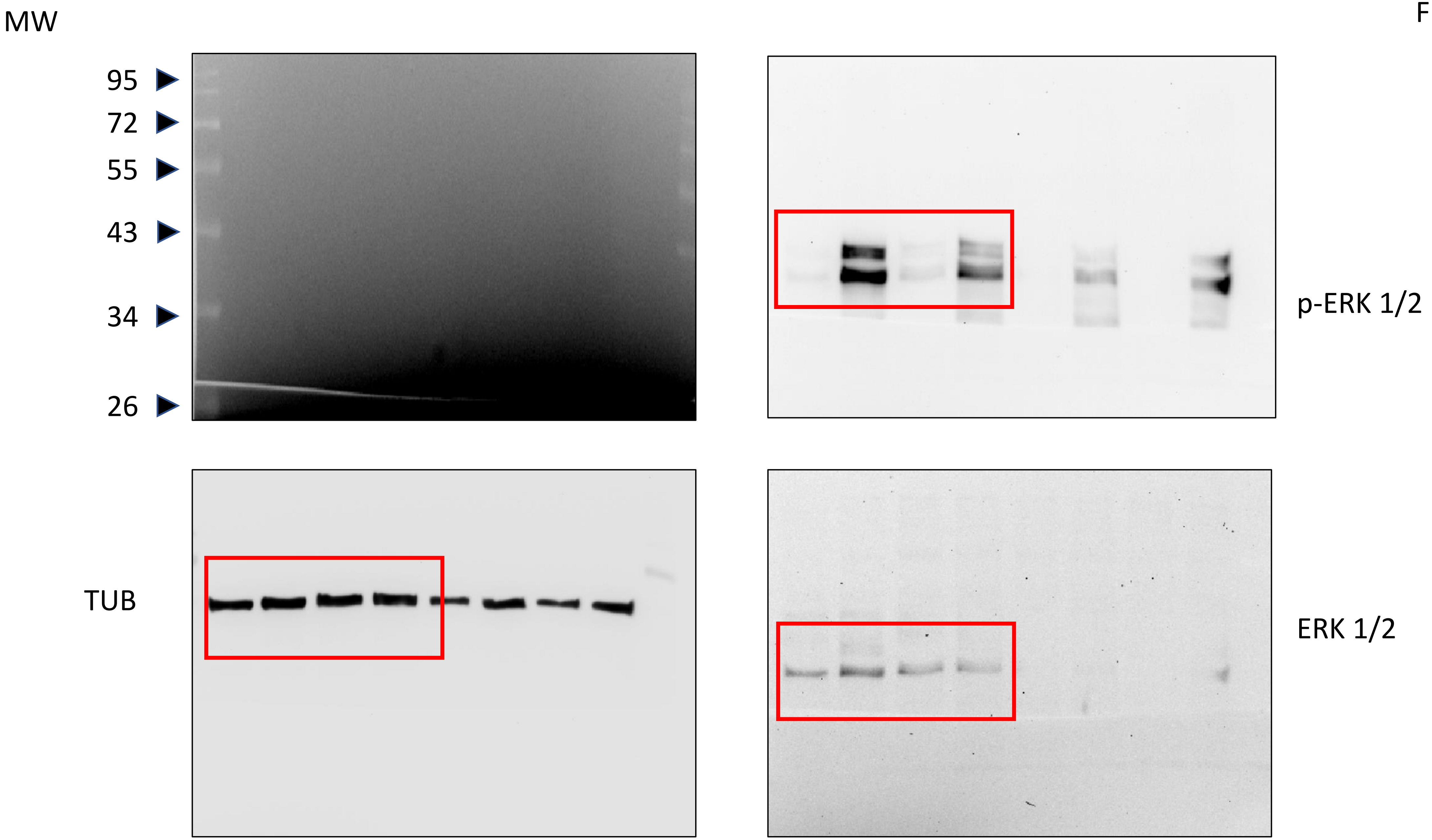

Figure 8A

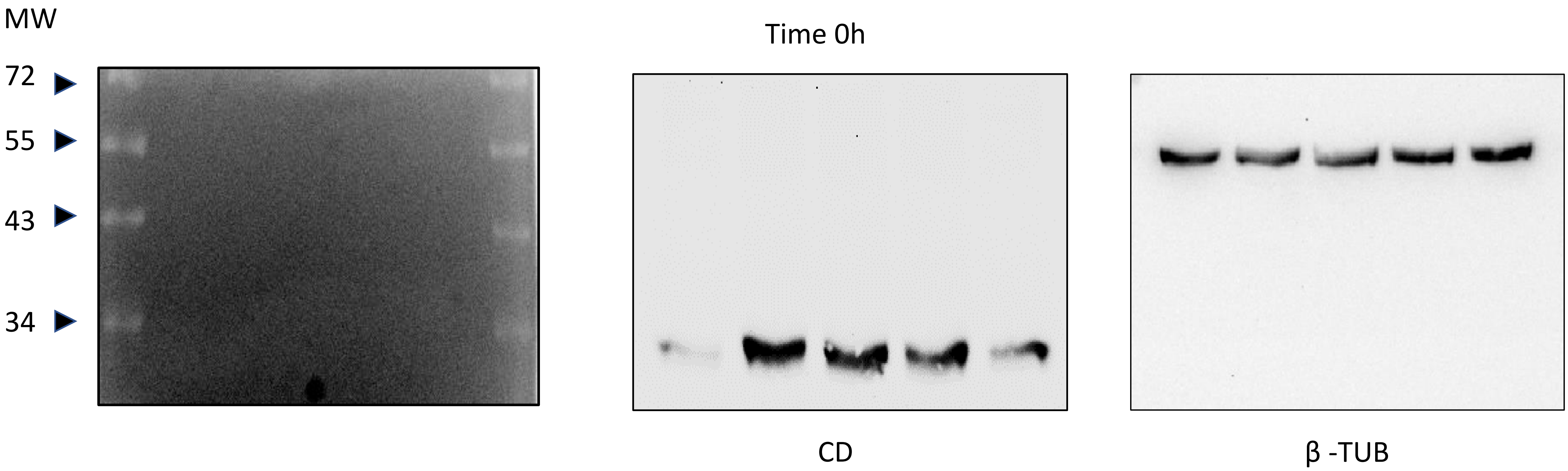

Figure 8B

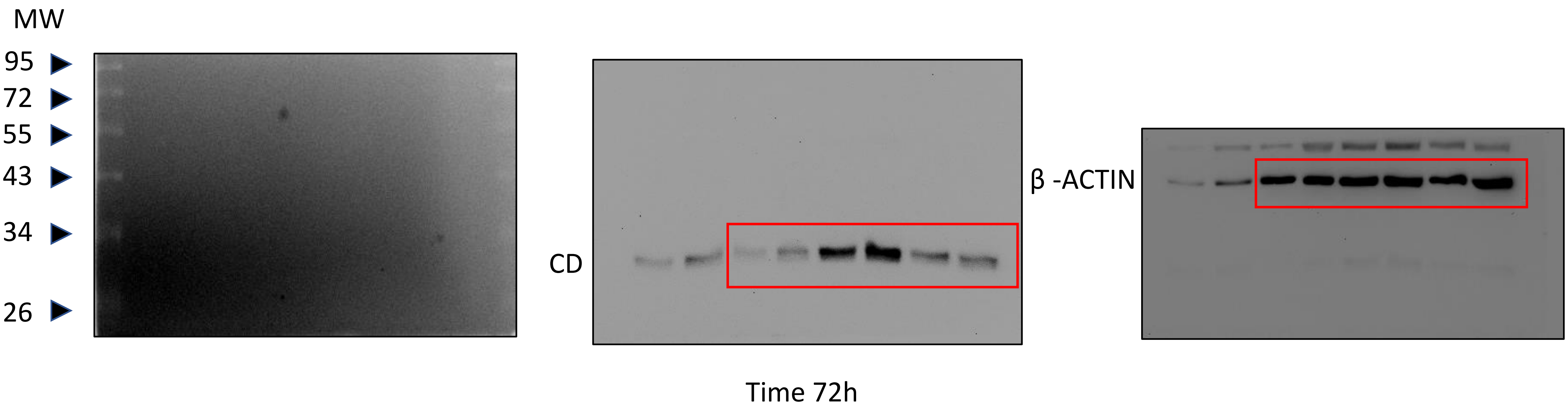

Figure 8C

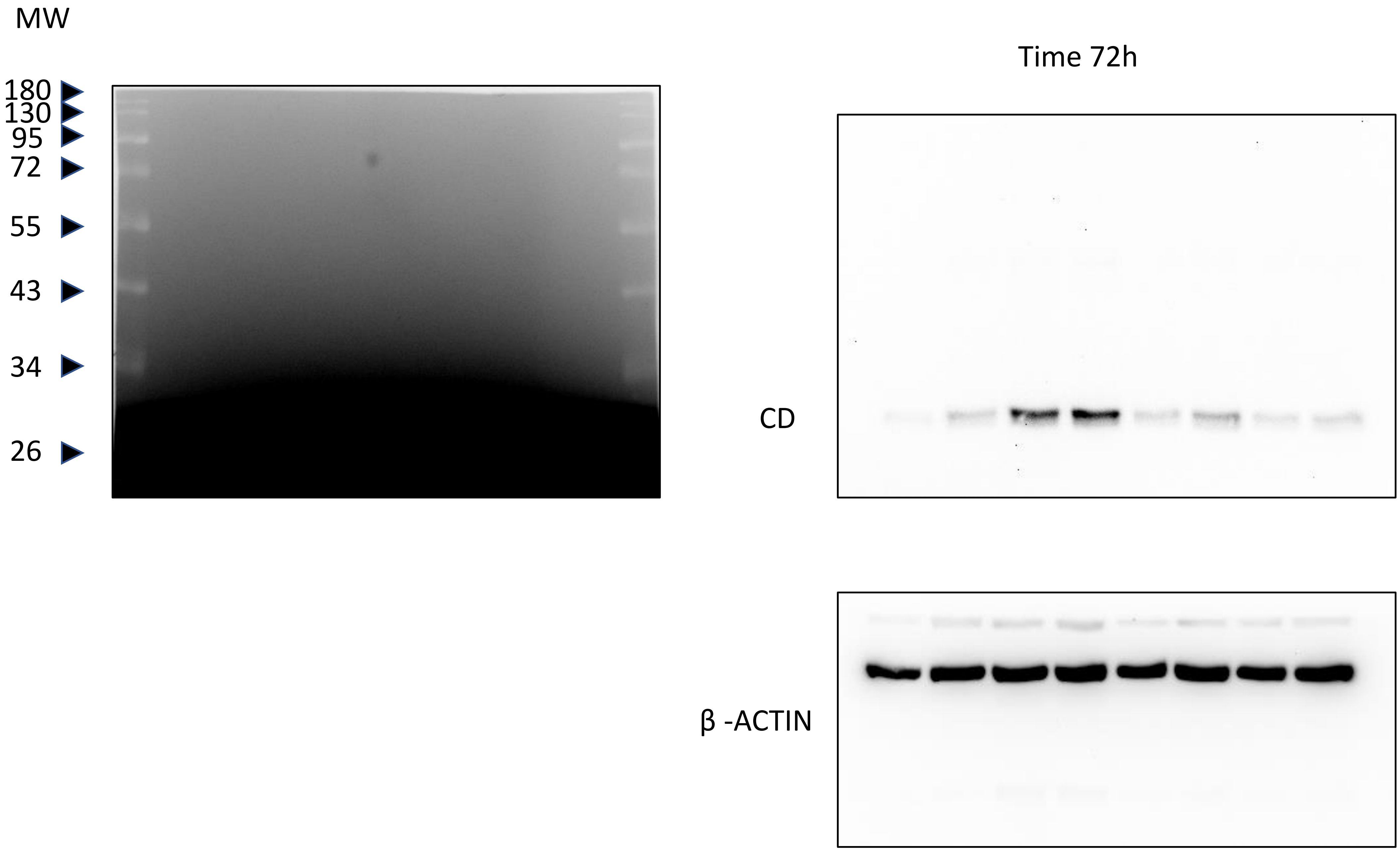

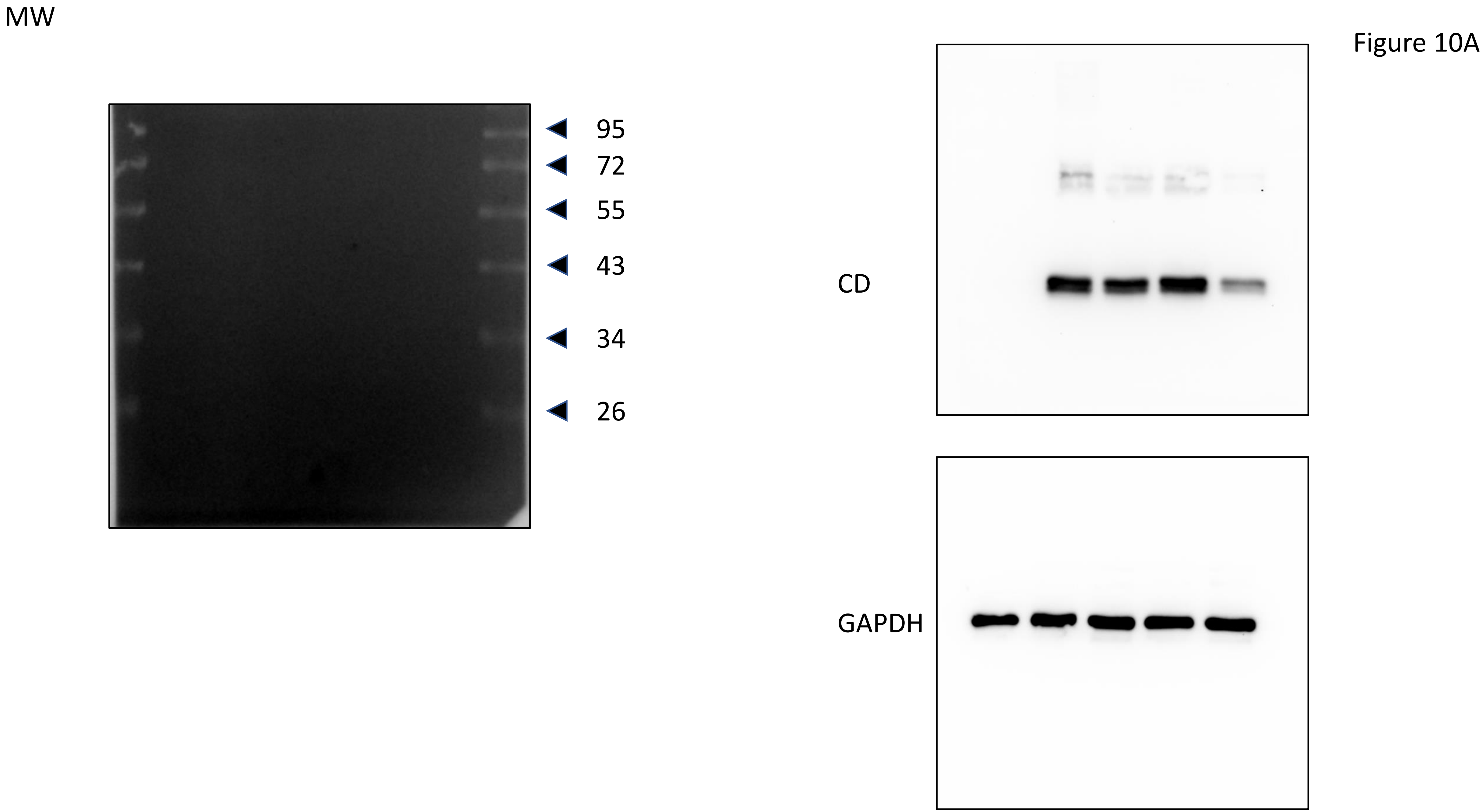
MW
95
72
55
43
34
26
CD
GAPDH
Figure 10A

Figure 10B

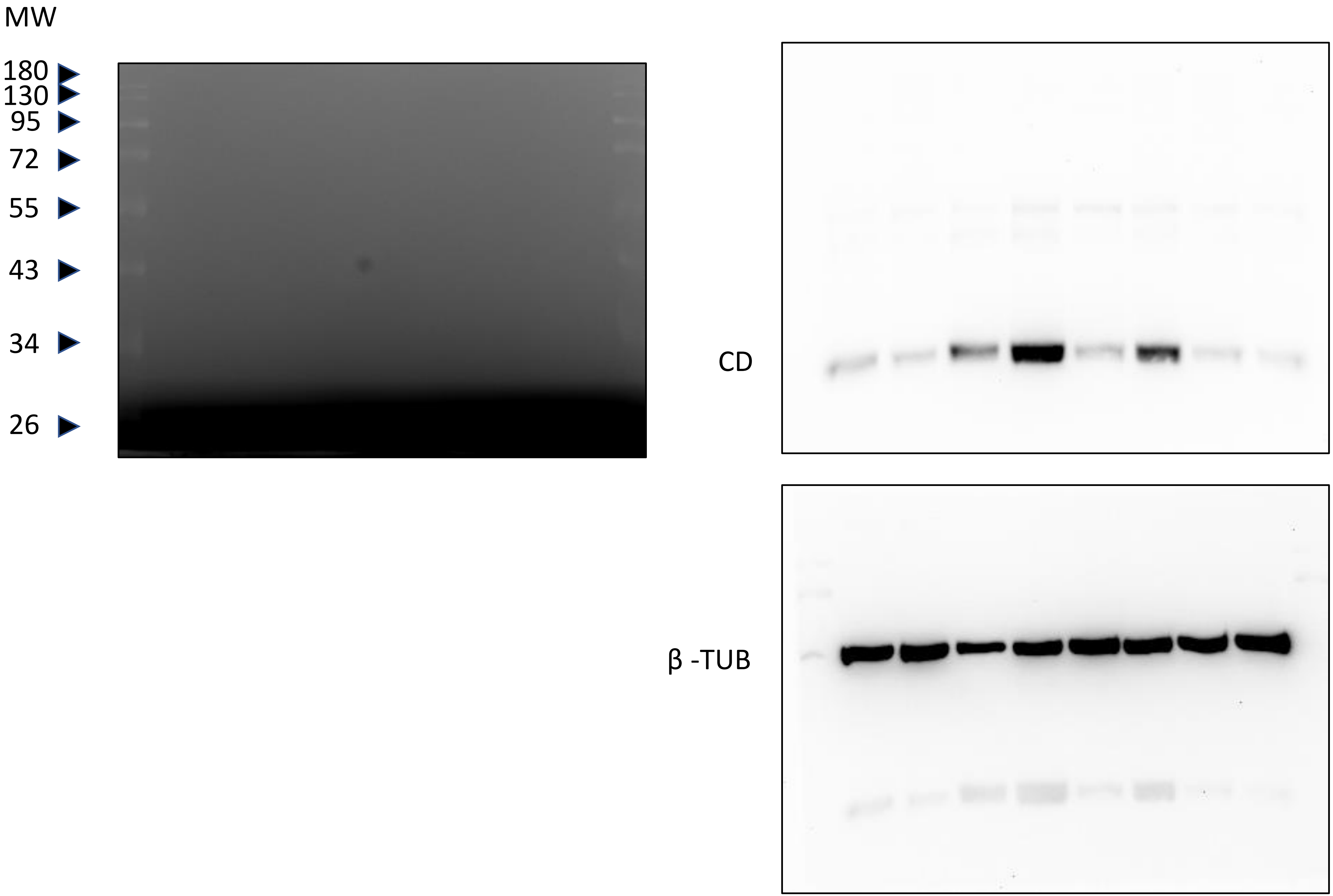

Ratio 1:1

Figure 10B

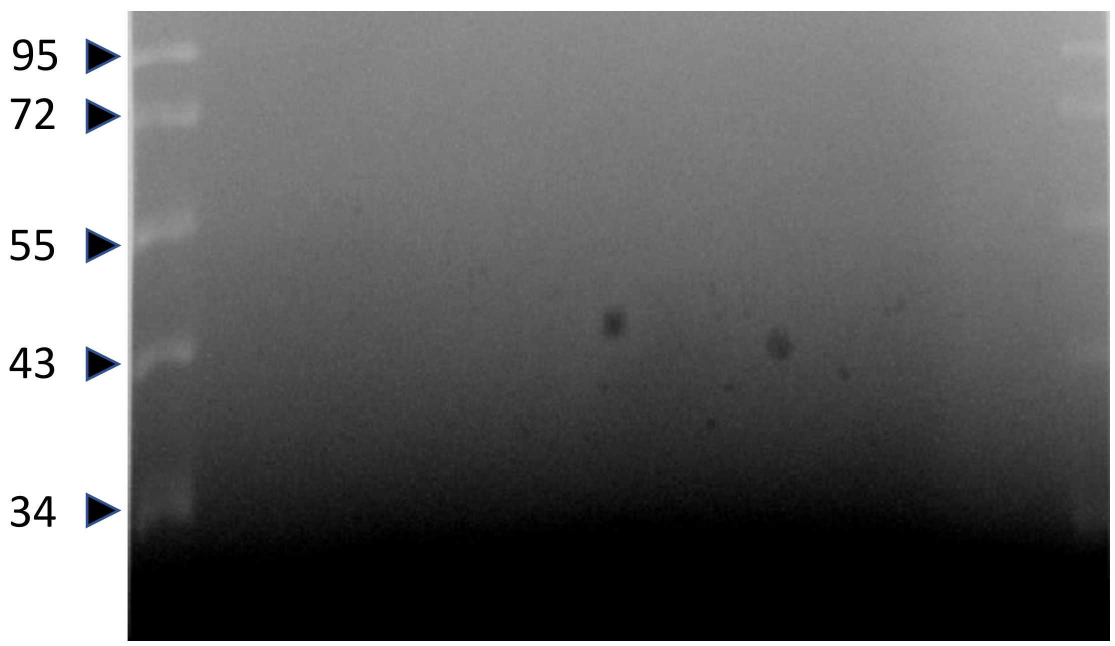

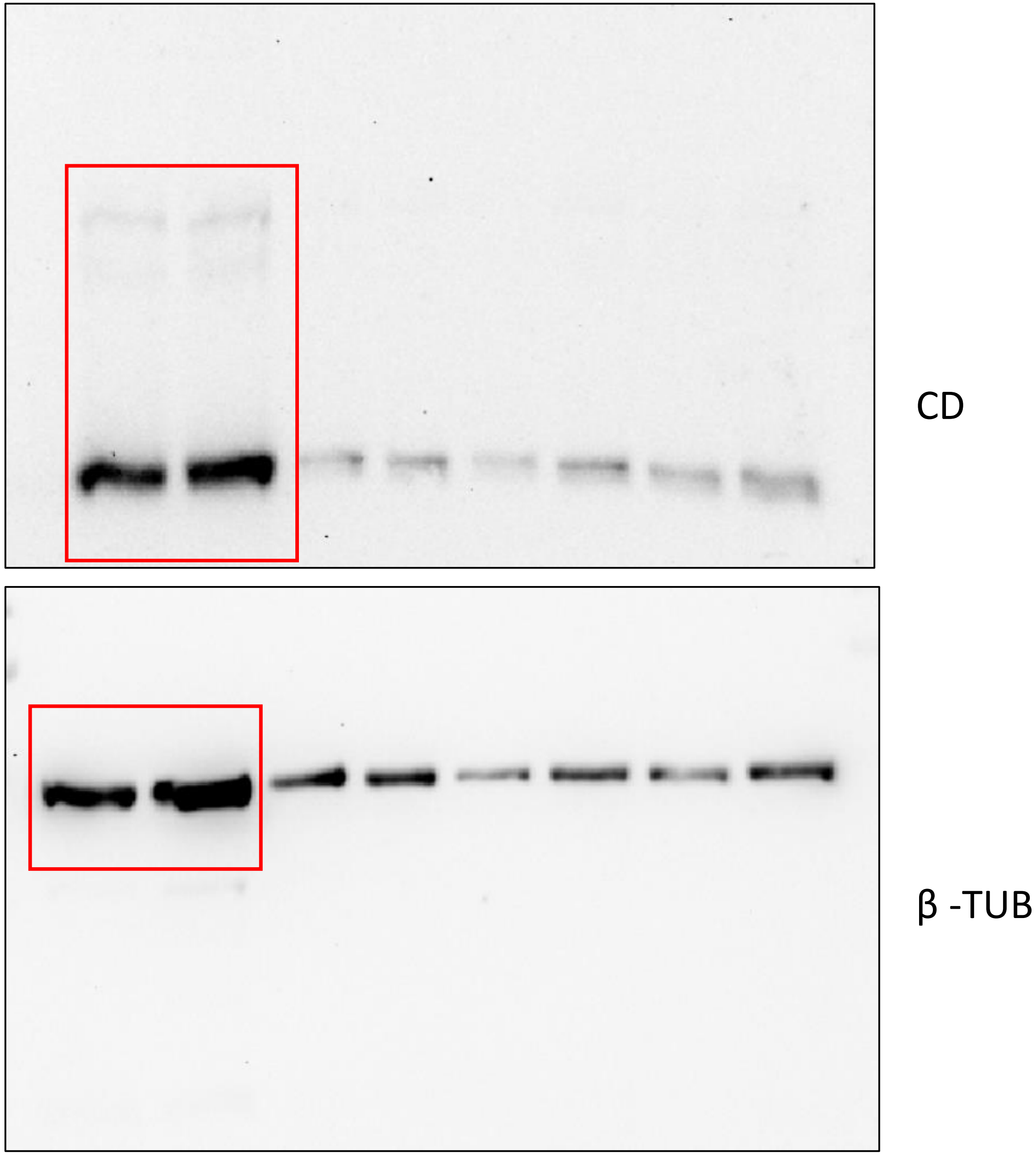

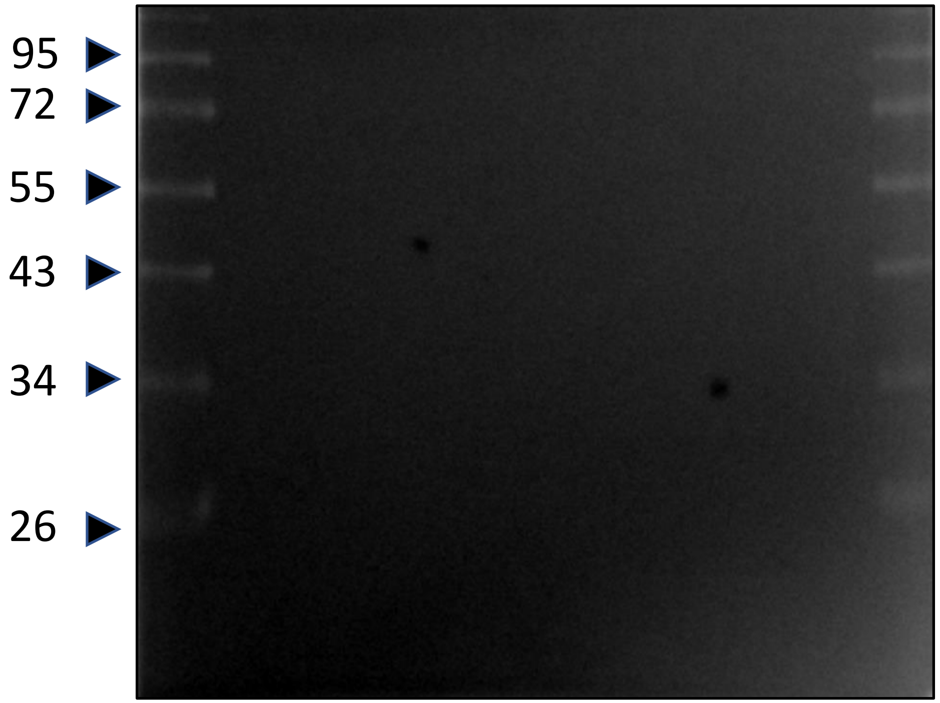

MW
95
72
55
43
34
26

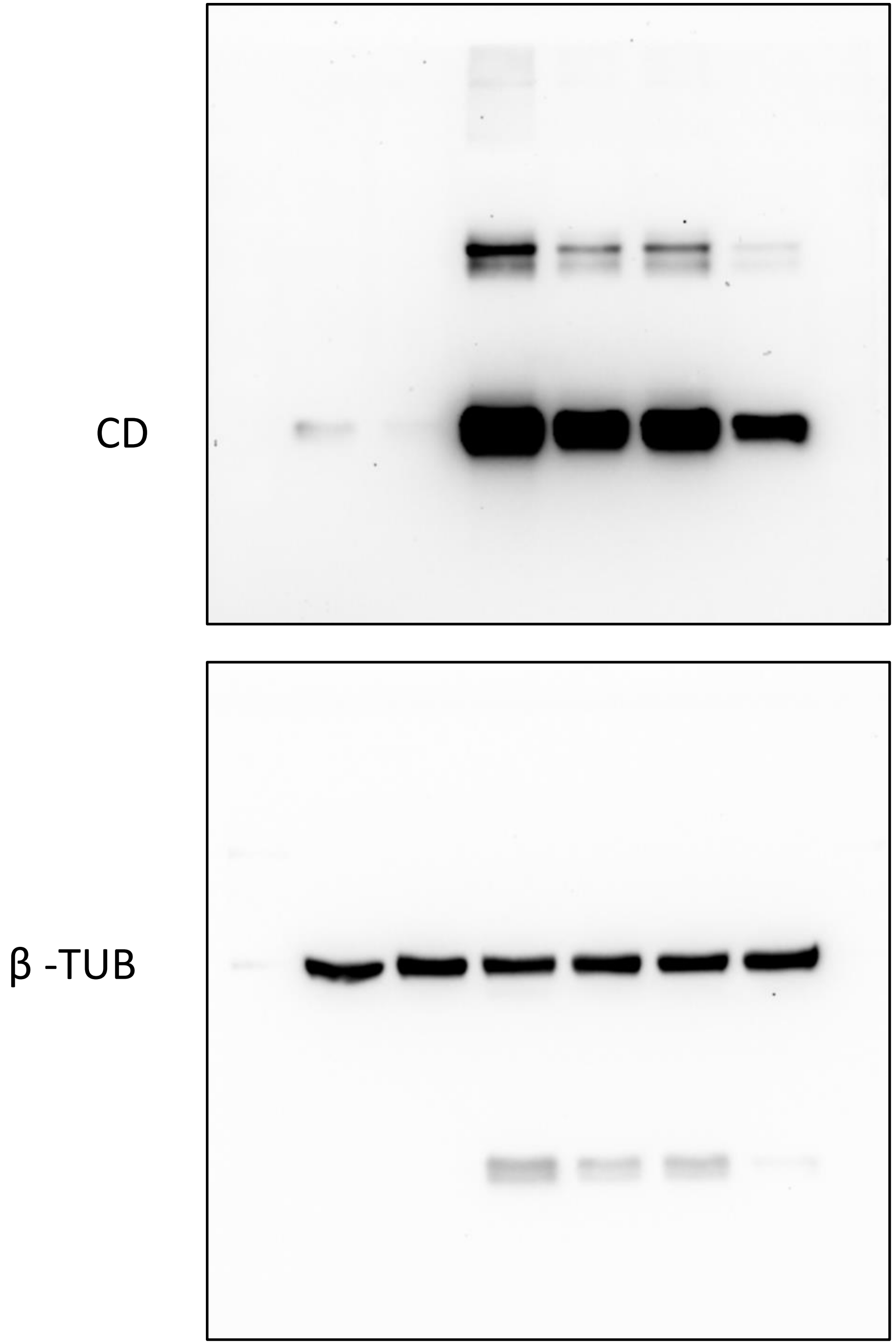

Figure 11C
CD
β -TUB

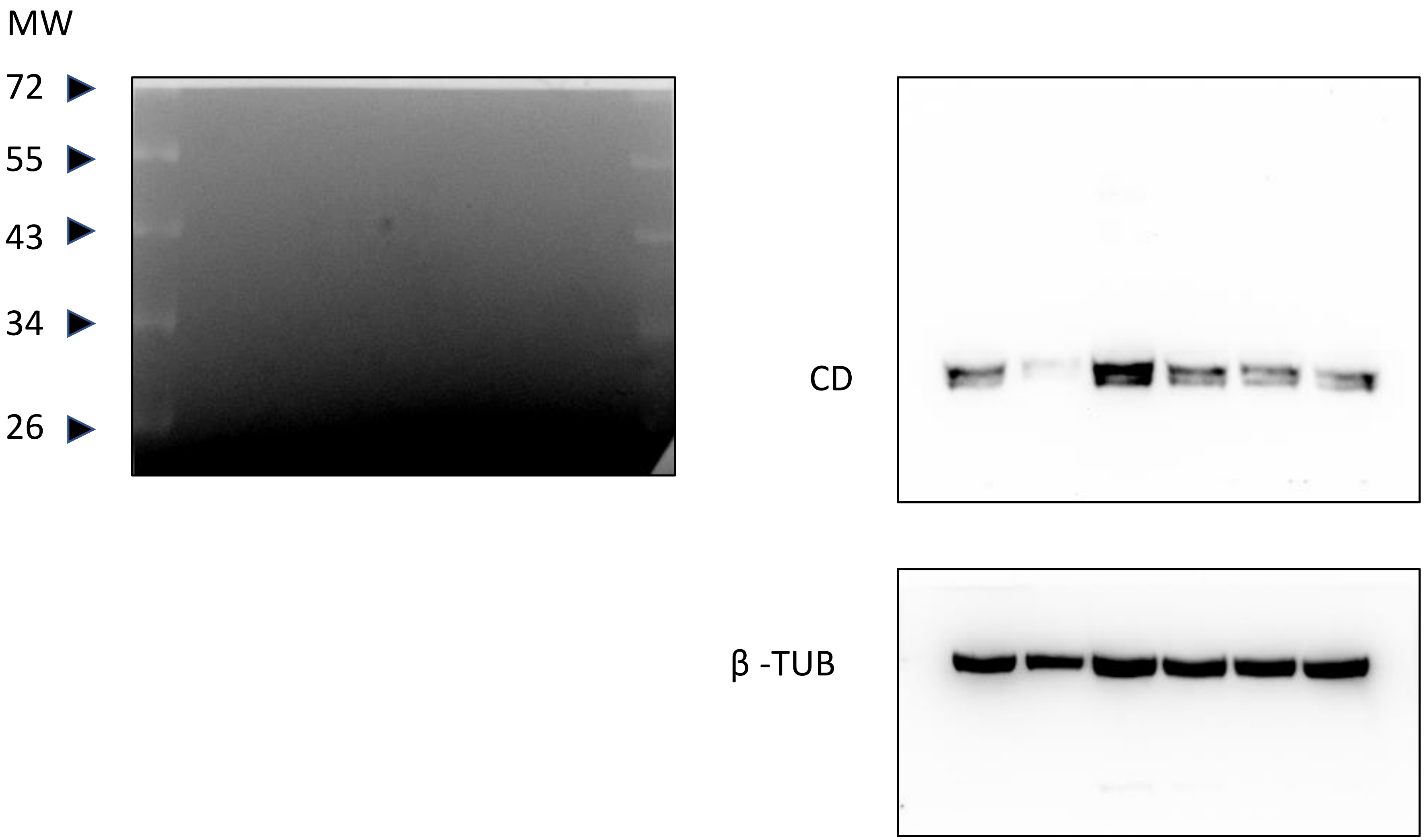

Figure 11D

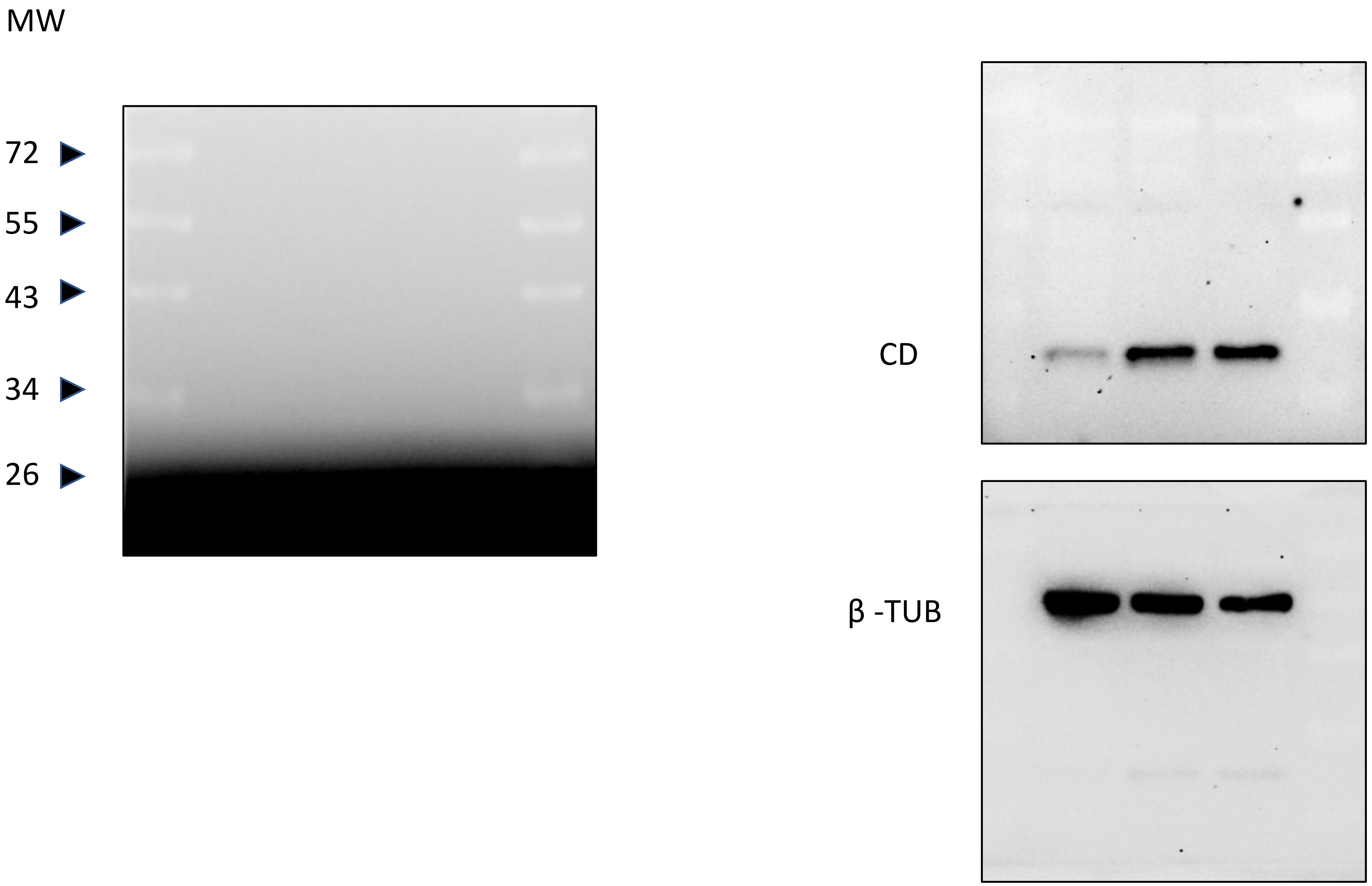

Figure 12C

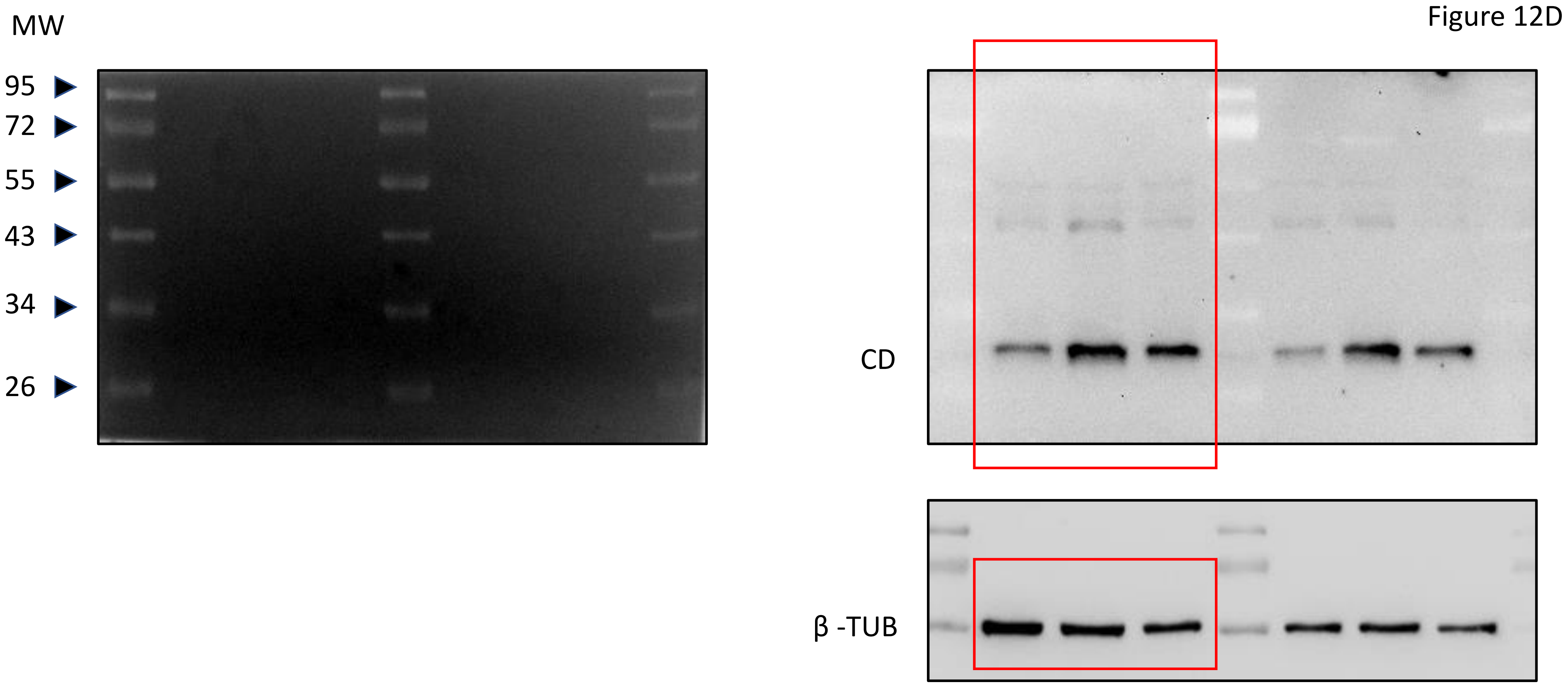
Figure 12D
MW
95
72
55
43
34
26
CD
β -TUB

Supplementary Figure S3 A

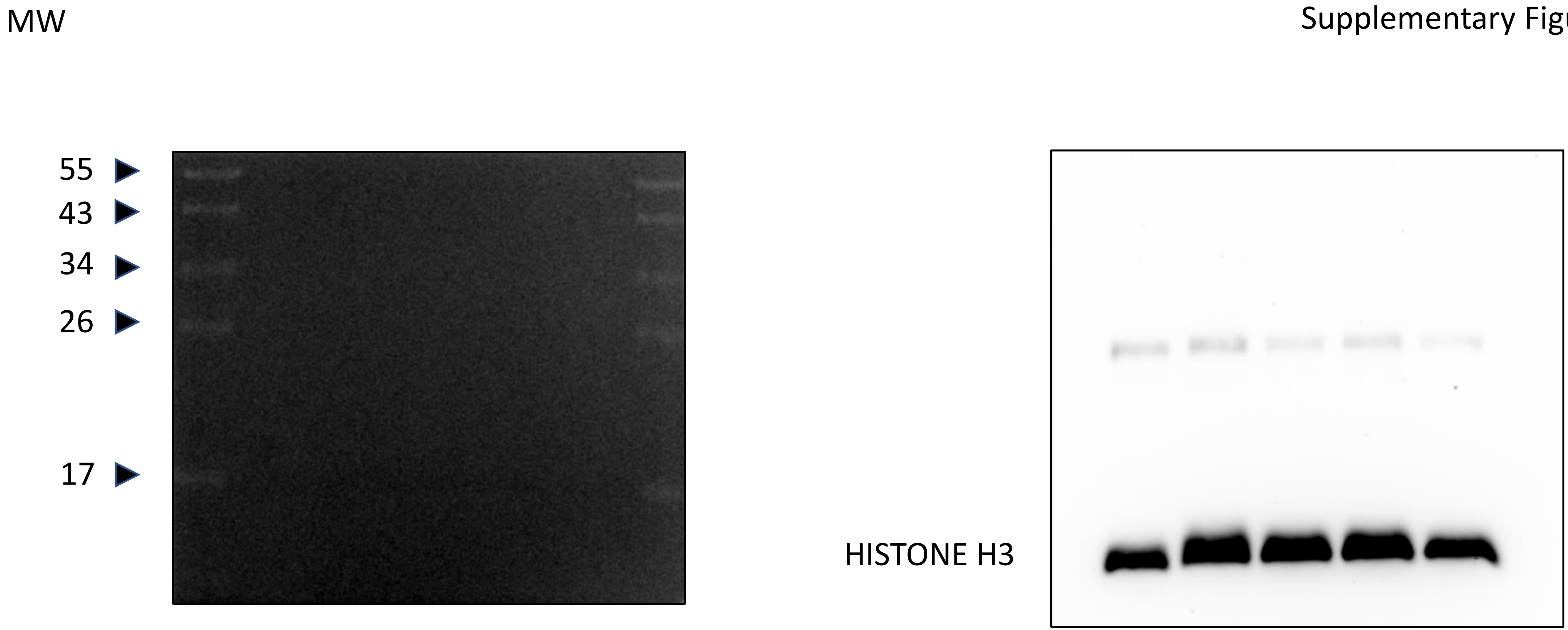

Supplementary Figure S3 B

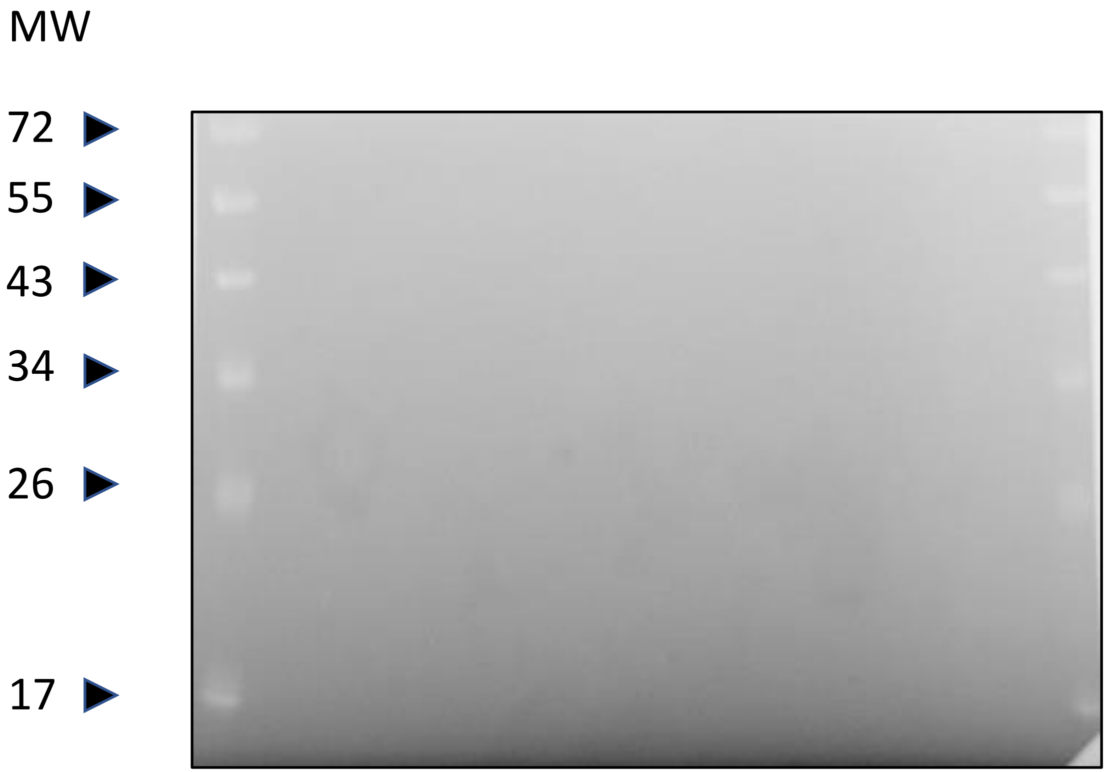

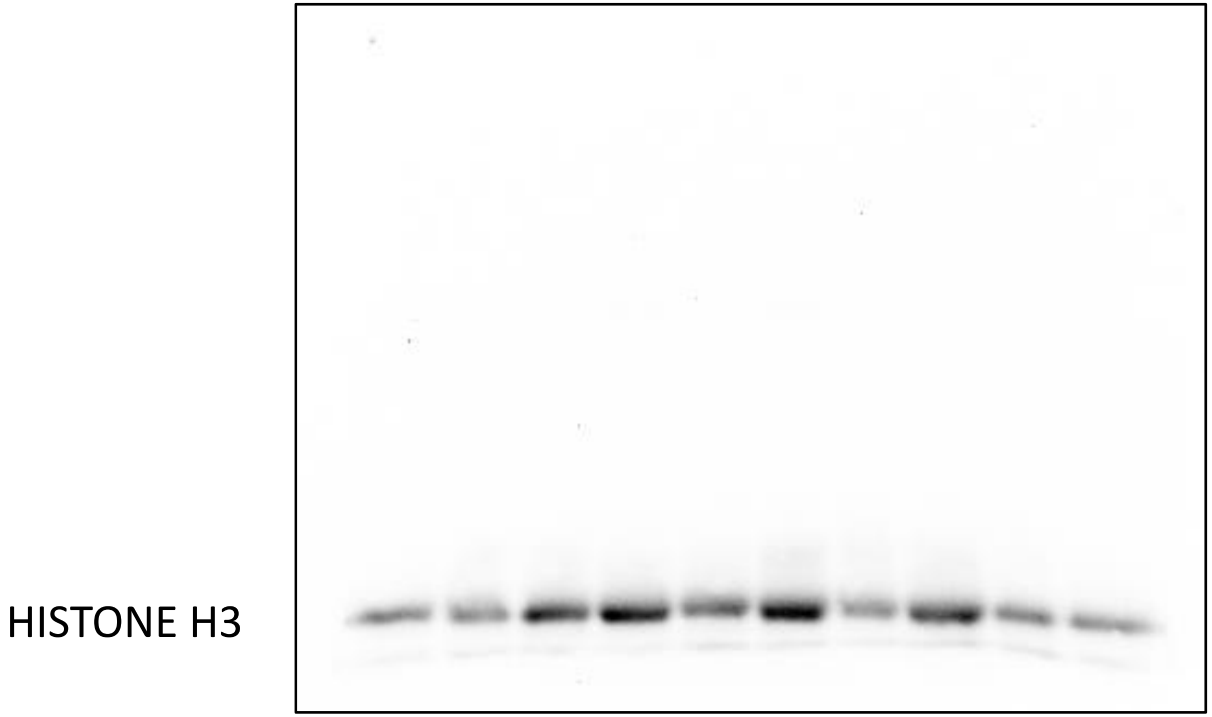

Supplement: Supplementary file 1 [file cancers-16-01343-s001.zip › Original Western blots.pdf]
